# Protocadherins at the Crossroad of Signaling Pathways

**DOI:** 10.3389/fnmol.2020.00117

**Published:** 2020-06-30

**Authors:** Anna Pancho, Tania Aerts, Manuela D. Mitsogiannis, Eve Seuntjens

**Affiliations:** Laboratory of Developmental Neurobiology, Department of Biology, KU Leuven, Leuven, Belgium

**Keywords:** clustered protocadherin, non-clustered protocadherin, WAVE, Wnt, apoptosis, cell adhesion, neural development

## Abstract

Protocadherins (Pcdhs) are cell adhesion molecules that belong to the cadherin superfamily, and are subdivided into clustered (cPcdhs) and non-clustered Pcdhs (ncPcdhs) in vertebrates. In this review, we summarize their discovery, expression mechanisms, and roles in neuronal development and cancer, thereby highlighting the context-dependent nature of their actions. We furthermore provide an extensive overview of current structural knowledge, and its implications concerning extracellular interactions between cPcdhs, ncPcdhs, and classical cadherins. Next, we survey the known molecular action mechanisms of Pcdhs, emphasizing the regulatory functions of proteolytic processing and domain shedding. In addition, we outline the importance of Pcdh intracellular domains in the regulation of downstream signaling cascades, and we describe putative Pcdh interactions with intracellular molecules including components of the WAVE complex, the Wnt pathway, and apoptotic cascades. Our overview combines molecular interaction data from different contexts, such as neural development and cancer. This comprehensive approach reveals potential common Pcdh signaling hubs, and points out future directions for research. Functional studies of such key factors within the context of neural development might yield innovative insights into the molecular etiology of Pcdh-related neurodevelopmental disorders.

## Introduction

Selective intercellular adhesion and cell-cell communication are key mechanisms for the proper development of organisms. Cell adhesion is mediated by different types of transmembrane molecules, of which the most prominent are the cadherins (Hirano and Takeichi, 2012). These are calcium-dependent cell adhesion proteins, a characteristic that led their discoverer, Masatoshi Takeichi, to coin in 1988 their name as a portmanteau of “calcium adherens” (Takeichi, 1988). The cadherin superfamily comprises several subfamilies, including the classical cadherins (type I and type II), desmosomal cadherins, protocadherins, flamingo/CELSR and cadherin related proteins, that all contain multiple cadherin motifs within their extracellular domain (Hulpiau and van Roy, 2009).

In this review, we will exclusively focus on protocadherins (Pcdhs). Several excellent reviews have covered the diverse roles that Pcdhs play in development (Redies et al., 2005; Yagi, 2013; Hayashi and Takeichi, 2015; Light and Jontes, 2017; Mountoufaris et al., 2018) and disease (Kahr et al., 2013; Hirabayashi and Yagi, 2014; Keeler et al., 2015a; El Hajj et al., 2017; Peek et al., 2017). Nevertheless, the way Pcdh engagement translates cell-cell interaction information in these different contexts to the cell remains elusive. After briefly describing the discovery, the characteristics, and the main roles of these Pcdhs, this review covers recent structural studies, molecular processing and downstream signaling in the context of cancer and neurodevelopment.

### History and General Characteristics of Pcdhs

The discovery of Pcdhs dates back to 1993, when several novel cadherin-like sequences were identified in a variety of organisms. Sano et al. described these molecules as similar to cadherins, but containing six or seven instead of five cadherin repeats in their ectodomain, as well as a transmembrane domain and a peculiar cytoplasmic tail. Indeed, the latter did neither show homology to the typical cadherin cytoplasmic tail nor complete conservation between different Pcdhs (Sano et al., 1993). Since the novel molecules somewhat resembled the Drosophila cell-adhesion protein Fat, Sano et al. suggested that the identified cadherin repeats could be derived from one primordial cadherin sequence, thus named the new molecules “protocadherins” (Sano et al., 1993). Comparing the evolutionary conservation of different Pcdhs, Hulpiau and van Roy suggested that they derived from an ancestral FAT-like cadherin by stepwise loss of extracellular cadherin (EC) repeats (Hulpiau and van Roy, 2009).

**FIGURE 1:**
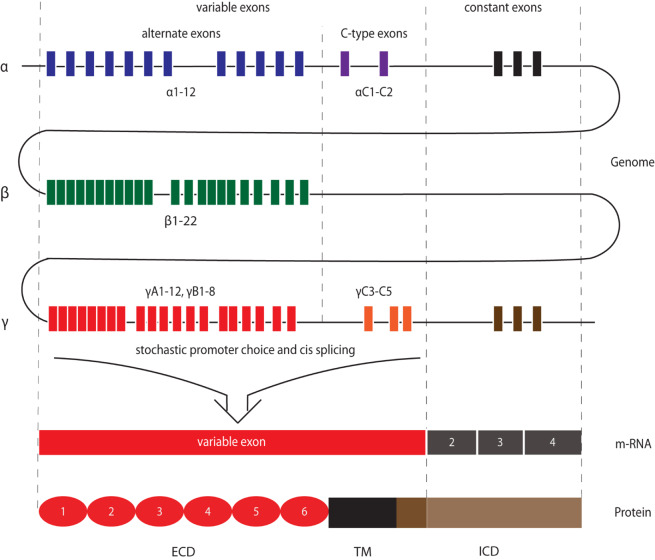
Schematic depiction of cPcdh gene organization to molecular structure. Both variable exons and constant exons encode cPcdhs. Variable exons are located upstream of constant exons for each cluster and are further categorized into alternate exons and C-type exons. The three constant exons in the α- and γ-Pcdh loci encode the common part of the respective ICDs. The β-Pcdh gene cluster does not encode C-isoforms nor presents constant exons, and therefore all related molecules lack the common ICD. Within the γ-Pcdh cluster alternate exons can be further subdivided into A- and B-type exons. After stochastic promoter choice and *cis* splicing, one variable exon encodes the extracellular domain (ECD), the transmembrane domain (TM), and part of the intracellular domain (ICD) of one Pcdh isoform. C-type exons encode C-isoforms.

**FIGURE 2:**
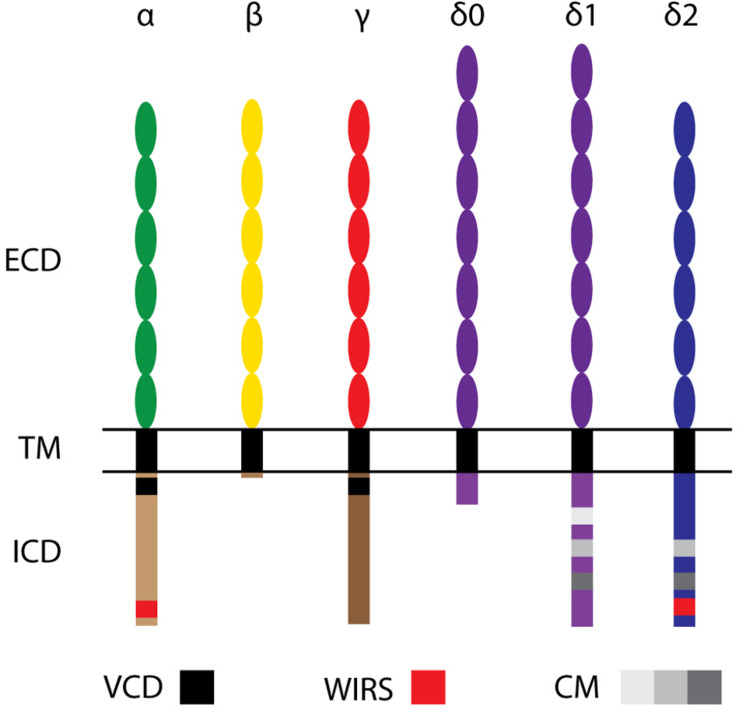
Molecular structure of Protocadherin family members. cPcdhs: α-, β-, and γ-Pcdhs present 6 extracellular cadherin (EC) repeats (ellipses) in their extracellular domain (ECD), a transmembrane domain (TM), and a conserved intracellular domain (ICD) (with the exception of β-Pcdhs, which possess a truncated ICD). The variable cytoplasmic domain (VCD) motif has been observed in some γ-Pcdhs and α-Pcdhs. ncPcdhs (δ0-Pcdhs, δ1-Pcdhs, and δ2-Pcdhs) represent transmembrane proteins with either 7 (for δ0-Pcdhs, δ1-Pcdhs) or 6 (for δ2-Pcdhs) EC repeats. Within their ICDs, δ1-Pcdhs have three conserved motifs (CM), while δ2-Pcdhs have two CMs. Moreover, δ2- and a few α-Pcdhs harbor a WAVE interacting receptor sequence (WIRS).

After being found to be expressed predominantly in mouse neural tissues and neuroblastoma cell lines, Pcdhs were independently discovered in 1998 as Cadherin-related neuronal receptors (CNRs) (Kohmura et al., 1998). Their expression at synaptic complexes suggested a possible role in establishing synaptic connections. CNRs were found to be encoded by clusters of tandemly arrayed genes and became known as clustered Pcdhs (cPcdhs). Furthermore, as cells expressed different combinations of a set of CNR variable exons, Kohmura et al. suggested that these molecules might form hetero-multimers that could equip cells with thousands of unique recognition modules (Kohmura et al., 1998). One year later, Wu and Maniatis found numerous additional CNR-like molecules encoded by tandemly arranged gene arrays, which were organized as three clusters (α, β, and γ) on human chromosome 5q31 (Wu and Maniatis, 1999). Due to their similarities to PCDH2 (Sano et al., 1993), they were included in the Pcdh family and subdivided according to their cluster in α-, β-, and γ-Pcdhs (Pcdh-α, Pcdh-β, Pcdh-γ) (Wu and Maniatis, 1999). Further sequence alignments and protein analyses revealed that these cPcdhs consist of a variable extracellular domain (ECD), a transmembrane domain (TM), and an intracellular domain (ICD) (Wu and Maniatis, 1999). For all three gene clusters, the ECD, the TM and a short part of the ICD are encoded by one large variable exon, a short part of the ICD are encoded by one large variable exon, while remainder of the ICD is encoded by three constant exons that are shared within a cluster (Wu and Maniatis, 2000) (Figure 1). Within the α- and γ-Pcdh clusters, the variable exons region can be further subdivided into alternate exons and C-type exons. Alternate exons can be classified into A- and B-type exons within the γ-Pcdh cluster. C-type exons are more similar to each other than to those encoding alternate isoforms within the same cluster, and generate α-Pcdhs C-isoforms C1 and C2, and γ-Pcdhs isoforms C3, C4, and C5 (Wu and Maniatis, 1999; Wu et al., 2001; Wang et al., 2002a). The β-Pcdh cluster lacks the constant exons and therefore encodes proteins with a truncated intracellular domain (Figure 2).

cPcdhs are generally conserved across vertebrate species, although the β-Pcdh cluster is missing in fugu, zebrafish and *Xenopus*, and the number of variable exons is not constant (Wu and Maniatis, 1999; Wu et al., 2001; Yu et al., 2007; Etlioglu et al., 2016).

At the time of their discovery, it was known that cPcdhs were not the only Pcdh subfamily members. Indeed, a number of *Pcdh* genes had been found to be scattered throughout the genome (Frank and Kemler, 2002; Redies et al., 2005; Vanhalst et al., 2005). The largest group of these non-clustered Pcdh (ncPcdh), the δ-Protocadherins (δ-Pcdhs), was identified via phylogenetic analysis. δ-Pcdhs can be further subdivided into δ 0-, δ1- and δ2-type based on their mutual homology and the number of ECD cadherin repeats (respectively, 7 and 6) (Vanhalst et al., 2005; Hulpiau and van Roy, 2009). Pcdh20 is the only δ0-Pcdh member (Hulpiau and van Roy, 2009). Members of the δ1-Pcdh subfamily include Pcdh1, Pcdh7, Pcdh9, and Pcdh11-X/-Y; members of the δ2-Pcdh subfamily are Pcdh8, Pcdh10, Pcdh17, Pcdh18 and Pcdh19 (Sano et al., 1993; Strehl et al., 1998; Hirano et al., 1999; Yoshida et al., 1999; Blanco et al., 2000; Ono et al., 2000; Wu and Maniatis, 2000; Wolverton and Lalande, 2001). δ-Pcdhs can have several isoforms, which contain identical extracellular domains, but differ in their cytoplasmic domain (Kim et al., 2011). While δ2-Pcdhs have two conserved motifs, CM1 and CM2, in their intracellular domain (Wolverton and Lalande, 2001), δ1-Pcdhs have an additional conserved motif (CM3) containing a putative binding site for protein phosphatase-1α (PP1α) (Vanhalst et al., 2005). Peculiarly, these conserved motifs are absent in other ncPcdhs: Pcdh12 and Pcdh20. Still, Pcdh20 has been classified as a δ0-Pcdh due to the strong homology of its 7 ECD to δ1-Pcdhs (Hulpiau and van Roy, 2009; Kim et al., 2011; Hulpiau et al., 2016).

Formerly, cadherin-related (Cdhr) proteins were considered as either Pcdhs or cadherins, although they have a distinct molecular structure and have evolved differently from both. They are related to cadherins as they present (at least two) consecutive EC repeats in their ECD. Some known misnomer examples are Pcdh15, Pcdh16, and μ-Pcdh. Based on additional comparative genomic analyses across metazoan organisms evolution they were later named Cdhr15, Cdhr6, and Cdhr5, respectively (Hulpiau and van Roy, 2009; Hulpiau et al., 2016; Gul et al., 2017).

## Expression and Roles of Pcdhs

Several ncPcdhs and cPcdhs are expressed most prominently within the central nervous system (Vanhalst et al., 2005; Redies et al., 2008; Kim et al., 2011; Hertel et al., 2012), which suggests important neurobiological roles for these molecules. On the other hand, loss of Pcdhs has been linked to several cancer types. In this section we summarize expression modalities of Pcdhs and, in relation to them, describe their roles in the nervous system and in cancer.

### Clustered Pcdhs in the Nervous System

#### Combinatorial Expression of cPcdh Isoforms Generates Cell Surface Diversity and Specificity

Expression studies of γ-Pcdh isoforms across subgroups (PcdhγA, PcdhγB, and PcdhγC) show generally overlapping patterns in large brain areas. While broader regions can express similar subsets of *Pcdhα* and *Pcdh*γ, alternative promoter selection and pre-mRNA *cis* splicing are used to generate specific combinations of different isoforms within individual cells (Tasic et al., 2002; Wang et al., 2002a).

Single cell RT-PCR analysis of Purkinje cells has revealed that most isoforms of these cPcdhs are monoallelically and combinatorially expressed in single neurons, whereas all five C-type isoforms are expressed biallelically and uniformly in all of these neurons (Esumi et al., 2005; Kaneko et al., 2006; Hirano et al., 2012). In contrast, C-type isoforms have been only found in a small percentage of mouse olfactory sensory neurons (OSN). Interestingly, immature OSN still express alternate and C-type isoforms, suggesting downregulation of C-type isoforms throughout their maturation (Mountoufaris et al., 2017). Studies performed on serotonergic neurons revealed an exclusive expression of Pcdh C-isoforms in these cells, with PcdhαC2 being the most prominently expressed (Chen W. V. et al., 2017; Katori et al., 2017).

Isoform expression thus seems to be cell type specific, and bound to complex regulatory mechanisms. CPcdhs expression level and specificity are also epigenetically regulated. Each variable exon contains a specific promoter that is regulated by its position within the cluster (Noguchi et al., 2009; Kaneko et al., 2014), the orientation of enhancer elements (Guo et al., 2015) and the DNA methylation status (Guo et al., 2012). Epigenetic regulation of promoter choice and alternative transcripts therefore immensely increases the diversity of cPcdhs that can be generated. For additional detailed information on the epigenetic regulation of *Pcdh*-α and *Pcdh-*γ gene expression we refer to recent excellent reviews (El Hajj et al., 2017; Mountoufaris et al., 2018; Canzio and Maniatis, 2019).

Collectively, these studies indicate that transcriptional regulation can generate a large cell-surface molecular diversity and specificity within single neurons, creating functional diversification (Esumi et al., 2005; Kaneko et al., 2006; Hirano et al., 2012; Chen W. V. et al., 2017; Katori et al., 2017; Mountoufaris et al., 2017). However, not all neuronal cell types express multiple cPcdhs, and expression can be dynamic during development. More careful mapping of expression at the single-cell level would reveal whether expression patterns are stochastic within certain cell populations or not.

#### Roles in Development of Dendrites and Synapses

Various members of all three Pcdh clusters localize on the neuronal soma, on dendrites and axons, and at growth cones and synapses in differentiating and mature neurons (Kohmura et al., 1998; Wang et al., 2002a; Kallenbach et al., 2003; Phillips et al., 2003; Junghans et al., 2008).

In the context of dendrite development, cPcdhs play important roles in dendritic self-avoidance. γ-Pcdh isoform diversity is essential for the discrimination between isoneural and heteroneural dendrites in retinal starburst amacrine cells (SAC)s, as loss of this diversity impairs dendritic self-avoidance (Lefebvre et al., 2012; Kostadinov and Sanes, 2015). In the cerebral cortex, γ-Pcdhs promote dendritic arborization complexity in layer V pyramidal neurons (Garrett et al., 2012). Recently it was shown that α- and γ-Pcdhs can functionally interact and cooperate in dendritic development in a context-dependent manner, and that together they mediate dendrite self-avoidance in Purkinje cells (Ing-Esteves et al., 2018).

cPcdhs are also implicated in spine morphogenesis. γ-Pcdhs negatively regulate mouse cortical dendritic spine morphogenesis *in vivo* (Molumby et al., 2017). In contrast, deletion or knockdown of the γ-Pcdh cluster has been associated with a reduction in spine density and dendritic complexity in mouse olfactory granule cells and cultured hippocampal neurons (Suo et al., 2012; Ledderose et al., 2013). CA1 pyramidal neurons and cultured hippocampal neurons of *Pcdha* null mutant mice display simple arbors and low dendritic spine densities. Knockdown of γ-Pcdhs and knockout of α-Pcdhs *in vitro* leads to similar defects as in *Pcdha* null mutant mice, suggesting that both γ- and α-Pcdh members contribute to dendritic arborization (Suo et al., 2012).

Although functional evidence is still lacking, the molecular diversity and isoform-specific homophilic binding properties of cPcdhs might provide a synaptic adhesive code to support synaptogenesis and proper neural connectivity (Kohmura et al., 1998; Serafini, 1999; Shapiro and Colman, 1999). α-Pcdhs are found in neocortical synapses (Kohmura et al., 1998) and in perisynaptic sites of preganglionic terminals in chicken (Blank et al., 2004). In mouse hippocampal neurons overexpression of a dominant-negative α-Pcdh ICD leads to a reduction in spine number and decrease of presynaptic synaptophysin (Suo et al., 2012). Whether α-Pcdh isoforms are involved in synaptic adhesion (Kohmura et al., 1998) as has been shown for γ-Pcdhs (Garrett and Weiner, 2009) remains to be elucidated. β-Pcdhs accumulate dendritically and post-synaptically in mammalian retinal and cerebellar neurons, suggesting a potential involvement of β-Pcdhs in synaptogenesis and synaptic refinement (Junghans et al., 2008; Puller and Haverkamp, 2011).

γ-Pcdhs are found in synaptic intracellular compartments such as axonal and dendritic tubulovesicular structures within some hippocampal neurons (Phillips et al., 2003; Fernández-Monreal et al., 2009). PcdhγC5 is localized in a subset of GABAergic and glutamatergic synapses in cultured hippocampal neurons, majorly at dendrites as shown by colocalization of PcdhγC5 with specific synaptic proteins such as GABAergic presynaptic glutamate decarboxylase and vesicular glutamate transporter 1 (Li et al., 2010). In addition, it has been shown that PcdhγC5 is important for the stabilization and maintenance of some GABAergic synapses, but not for their formation (Li et al., 2012a). γ-Pcdhs were shown as well to play a role in synaptic elimination between closely spaced SACs, and in preventing autapse formation. Functional connectivity was impaired in neighboring SACs expressing a single γ-Pcdh isoform, demonstrating a necessary function of isoform diversity in establishing inter-SAC networks (Kostadinov and Sanes, 2015). In conclusion, several lines of evidence point to γ-Pcdhs as key molecular players in synapse formation, stabilization and maintenance in the mammalian nervous system. Unfortunately, current knowledge is limited to particular cPcdhs and specific neuronal cell types, hence studies focusing on different neuronal types or α and β clusters in this context might be revealing in the future. However, even for γ-Pcdhs exact roles in the regulation of synaptic function remain to be defined, and are likely to be context-dependent.

#### Roles in Axonal Development, Targeting and Branch Repulsion

cPcdhs participate in several aspects of axonal development, and their potential to generate unique molecular codes are at the basis of both axon-target and axon-axon recognition mechanisms.

α-Pcdhs are indispensable for axon growth in cultured hippocampal neurons (Lu et al., 2018). Whether this role is unique to this cluster remains to be investigated. In the mouse spinal cord, loss of γ-Pcdhs leads to severe disorganization of Ia primary afferent projection terminals in the ventral horn, leading to a targeting defect between Ia afferents and ventral horn interneurons that suggests a critical role of γ-Pcdh-mediated recognition between the two (Prasad and Weiner, 2011; Hasegawa et al., 2016).

Other studies have revealed functions in axon targeting and branch repulsion which appear to be redundantly shared by all cPcdhs. Deletion of all three clusters, but not of single clusters, leads to the complete disruption of axonal arborization and to clumping of axonal terminals in mouse OSN. Overriding Pcdh diversity through overexpression of a fixed set of 3 cPcdhs (one α-, one β-, and one γ-Pcdh) in OSN results in the failure of axon terminals to converge and form normal glomeruli. In this case, the induced expression of an identical cPcdh membrane code seems to result in the erroneous self-avoidance between non-self axons (Mountoufaris et al., 2017).

In sharp contrast to what has been observed in the development of OSN connectivity, axonal tiling of serotonergic neurons is highly dependent on a single cPcdh isoform, PcdhαC2, that drives repulsion between neurites of distinct cells and ensures proper spatial axon distribution (Chen W. V. et al., 2017; Katori et al., 2017). To what extent other specific cPcdh isoforms have unique roles in the arrangement and targeting of projections between specific subpopulations of neuronal cells remains to be addressed.

#### Roles in Neuronal Survival

Members of the γ-Pcdh cluster are known to prevent neuronal apoptosis of spinal cord interneurons, retinal cells, and cortical interneurons (cINs) (Wang et al., 2002b; Lefebvre et al., 2008; Prasad et al., 2008; Chen W. V. et al., 2012; Garrett et al., 2012; Hasegawa et al., 2016, 2017; Carriere et al., 2020; Leon et al., 2020). Transplantation studies and knockout mice phenotyping showed that loss of C-type γ-Pcdhs leads to increased cell death in spinal cord interneurons and cINs (Chen W. V. et al., 2012; Leon et al., 2020). Remarkably, within the C-type γ-Pcdhs, only one isoform (PcdhγC4) seems to be necessary for neuronal survival of several cell populations (Garrett et al., 2019).

While γ-Pcdhs appear to be particularly important in mouse neuronal survival, in zebrafish truncation of an α-Pcdh, Pcdh1α, has been found to lead to neuronal cell death in the developing brain and spinal cord (Emond and Jontes, 2008).

Different cPcdh clusters can also cooperate in the regulation of cell death and survival. For instance, the α-Pcdh and γ-Pcdh clusters have been demonstrated to cooperatively regulate neuronal survival in the retina (Ing-Esteves et al., 2018). In the spinal cord, interneuron apoptosis is aggravated in βγ-Pcdh and αβγ-Pcdh deficient mice compared to mice lacking only γ-Pcdhs, suggesting this process to be cPcdh dosage-dependent (Hasegawa et al., 2016). Moreover, cPcdhs seem to cooperate to prevent apoptosis in a cell type-specific manner. In chimeric mice lacking all three clusters (αβγ-Pcdh deficient mice), survival rates were found to significantly decrease in neuronal populations in the midbrain, pons and medulla, but not in the inferior olive, in sensory and motor neurons, and neuronal populations within the cerebral cortex and olfactory bulb (Wang et al., 2002b; Hasegawa et al., 2016, 2017). Therefore, context-specific combinatorial expression of cPcdhs appears to be important in the regulation of neurodevelopmental cell death versus survival.

### Non-clustered Pcdhs in the Nervous System

#### Combinatorial Expression of ncPcdhs Contributes to Specification of Neuronal Identity

Similarly to classical cadherins, ncPcdh expression is spatiotemporally regulated during brain development in several vertebrate species. In zebrafish and chicken, this mode of expression characterizes transcription of *Pcdh9, Pcdh17*, and *Pcdh19* in the nervous system (Liu et al., 2009, 2010; Hertel et al., 2012; Lin et al., 2012). In the mouse brain, *Pcdh7*, *Pcdh9* and *Pcdh11* expression localizes to restricted regions within the neocortex, hippocampus and amygdala (Vanhalst et al., 2005). In rat, *Pcdh1*, *Pcdh9*, *Pcdh10*, *Pcdh17*, *Pcdh19*, and *Pcdh20* are specifically expressed in limbic system structures, such as the hippocampus, the limbic cortex, the thalamus, the hypothalamus, and the amygdala. Cortical region-dependent and layer-specific expression can also be observed perinatally (Kim et al., 2007). In addition, Pcdh10 synthesis has been described in specific networks like the limbic and visual systems in mice and chicken (Hirano et al., 1999; Aoki et al., 2003; Müller et al., 2004).

Besides being present in distinct brain areas, δ-Pcdhs have been demonstrated to be combinatorially expressed in the ferret retina and in the mouse primary sensory cortex (Etzrodt et al., 2009; Krishna-K et al., 2011). Moreover, it was recently shown that one mouse OSN can express up to seven δ-Pcdhs, and that cells can adjust the number and expression levels of δ-Pcdhs on their surface to regulate their adhesivity (Bisogni et al., 2018).

Overall, the combinatorial and molecule-specific spatiotemporal expression patterns of ncPcdhs in the vertebrate central nervous system point at roles in neural circuit formation, potentially by contributing to a molecular recognition code.

#### Roles in Synaptic, Dendritic, and Axonal Development

Like cPcdhs, δ-Pcdhs are localized in dendrites, axons, and proximally to or within synapses (Hirano et al., 1999; Yasuda et al., 2007; Hoshina et al., 2013; Pederick et al., 2016). All members of the δ1-Pcdh family and the majority of the δ2-Pcdh family, with the exception of Pcdh18, have been associated to the regulation of dendritic initiation, growth, morphology, arbor refinement, and spine formation (Yasuda et al., 2007; Piper et al., 2008; Bruining et al., 2015; Wu et al., 2015; Schoch et al., 2017; Bassani et al., 2018; Chang et al., 2018). To date, roles in axon growth, branching, guidance, and fasciculation have been described for Pcdh7 and for almost all of the δ2-Pcdhs except Pcdh8 (Aoki et al., 2003; Uemura et al., 2007; Nakao et al., 2008; Piper et al., 2008; Leung et al., 2013, 2015; Biswas et al., 2014; Hayashi et al., 2014; Cooper et al., 2015; Asakawa and Kawakami, 2018; Guemez-Gamboa et al., 2018). While specific synaptic roles have not yet been directly demonstrated for δ1-Pcdhs, they have been suggested to participate in synaptic maintenance and plasticity based on their expression at synapses and interaction with PP1α (Yoshida et al., 1999; Vanhalst et al., 2005; Kim et al., 2007; Bruining et al., 2015). Roles in synaptogenesis, synaptic vesicle assembly and mobility, synapse elimination, and synaptic connectivity have been demonstrated for all δ2-Pcdhs (Yasuda et al., 2007; Tsai et al., 2012; Hoshina et al., 2013; Biswas et al., 2014; Bassani et al., 2018; Light and Jontes, 2019; Lv et al., 2019). While the role of cPcdhs in neuronal self-avoidance has been extensively described (see sections above), nearly no information is available for ncPcdhs in this context. Interestingly, removal of the Pcdh17 cytoplasmic domain in zebrafish has been shown to induce axon clumping in somatic motor neurons, but not in spinal interneurons, pointing to cell-type specific roles in fasciculation and guidance/targeting via ncPcdh-mediated homotypic repulsion (Asakawa and Kawakami, 2018). Table 1 summarizes currently known roles of ncPcdhs in synapse, dendrite, and axon development.

**TABLE 1:** Different roles of ncPcdhs in dendritogenesis, axon development and synaptogenesis.

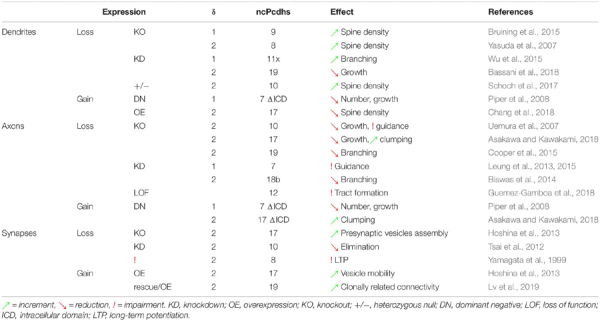

### Roles of Pcdhs in Cancer

In addition to the expression of Pcdhs during (brain) development, several tissues maintain expression of these molecules at adult stages. While research into their exact function at these stages is still in its infancy, they might regulate cellular differentiation, tissue regeneration and maintenance. Best studied in this regard is *PCDH1* in lung epithelial cells (Faura Tellez et al., 2015, 2016; Kozu et al., 2015). Consequently, dysregulation of *PCDH* expression has been extensively associated with multiple types of cancer. This reflects the wider-scale relationship between oncogenesis and cadherin-mediated cell adhesion, as members from other cadherin subfamilies are known to be involved in tumor suppression or progression (reviewed in detail by van Roy (2014). While most *PCDH* genes are considered tumor suppressors, multiple exceptions have been observed.

The epigenetic mechanisms governing the expression of *cPCDHs* and their unique genomic organization render them sensitive to long range epigenetic silencing (LRES). Indeed, agglomerative hypermethylation of all three or individual *cPCDH* clusters has been identified in breast cancer, cervical cancer, colorectal cancers, uterine leiomyosarcoma and leiomyoma, and in Wilms’ tumor (Novak et al., 2008; Dallosso et al., 2009, 2012; Miyata et al., 2015; Wang et al., 2015). Moreover, a vast array of studies has identified individual *PCDH* downregulation due to promoter hypermethylation or somatic aberrations (see Table 2). The most frequently reported phenotypes resulting from loss of *PCDH* expression are increased proliferation and decreased apoptosis; however, considering that these processes are the most commonly investigated in cancer, it is likely that additional loss of function effects have been understudied. Several signaling pathways have been linked to the regulation of proliferation (Wnt/β-catenin signaling and Pi3K/AKT-signaling) and apoptosis (NF-κB and DEPDC1-caspase signaling) by PCDHs in cancer (Hu et al., 2013; Li et al., 2014; Chen et al., 2015; Lv et al., 2015; Xu et al., 2015; Yang et al., 2016; Ye et al., 2017; Zong et al., 2017). Less commonly reported consequences of *PCDH* loss include increased migration/invasion, epithelial–mesenchymal transition, angiogenesis, and resistance to drugs. The signaling pathways that PCDHs utilize to regulate these processes are still poorly understood (Li et al., 2012b; Zhu et al., 2014; Chen et al., 2015; Lv et al., 2015). Loss of *PCDH* expression in cancer is often an indicator of poor prognosis, either directly as a result of metastasis, or indirectly through increased resistance to drugs and apoptosis (see references Table 2). Overall, downregulation of all classes of *PCDHs* in somatic cells has been associated with cancer malignancy, supporting a role in the control of cell survival, proliferation, and migration.

**TABLE 2:** Downregulation of Pcdh genes in cancer due to somatic aberrations (SA) or promoter hypermethylation (PH).

Organ	reg.	PCDH gene		References
Bladder	PH	*c*	*GA12*	Reinert et al., 2011
		nc	*8, 7, 17*	Costa et al., 2011; Lin et al., 2014a; Wang X. B. et al., 2014
Blood	PH	c	*GA12, GB7*	Shi et al., 2007; Taylor et al., 2007
		nc	*8, 10, 17*	Ying et al., 2007; Leshchenko et al., 2010; Narayan et al., 2011; Li et al., 2012b; Uyen et al., 2017
Brain	PH	*c*	*A8, A13, GC4, GA11*	Waha et al., 2005; Abe et al., 2008; Zhang et al., 2011
	both	nc	*10*	Bertrand et al., 2011
	SA	nc	*8, 9*	
Breast	PH	*c*	*GB6, B15*	Miyamoto et al., 2005; Zhang et al., 2015
		nc	*1, 10, 17*	Miyamoto et al., 2005; Vasilatos et al., 2013; Yin et al., 2016
	both	nc	*8*	Yu et al., 2008
Cervix and ovary	PH	nc	*10*	Narayan et al., 2009
	SA	nc	*9*	Shi et al., 2019
Colon and rectum	PH	c	*B3, GA7*	Ye et al., 2018; Liu et al., 2019
		nc	*10, 17, 18*	Hu et al., 2013; Zhong et al., 2013; Zhou D. et al., 2017
Esophagus, hypo-, and nasopharynx	PH	nc	*8, 17, 20*	Giefing et al., 2011; He et al., 2012; Chen et al., 2015
	both	nc	*10*	Ying et al., 2006
Kidney	PH	c	*B5*	Xia et al., 2017
		nc	*8*	Morris et al., 2011
Liver	PH	nc	*10, 19*	Ying et al., 2006; Fang et al., 2013; Zhang et al., 2018
	both	nc	*9*	
	SA	nc	*17, 20*	Lv et al., 2015; Dang et al., 2016
Lung	PH	c	*GB6*	Liu et al., 2018
		nc	*10, 17, 20*	Imoto et al., 2006; Ying et al., 2006; Um et al., 2017
Muscle	PH	c	*A4*	Tombolan et al., 2016
Pancreas	PH	c	*GA6, GB1, GB6*	Vincent et al., 2011
		nc	*1, 8, 10, 11Y*	Vincent et al., 2011; Qiu et al., 2016
	both	c	*GC4*	Jones et al., 2008; Vincent et al., 2011
		nc	*17*	Jones et al., 2008; Vincent et al., 2011
	SA	c	*B2, B16, GA1, GA11*	Jones et al., 2008
		nc	*9,18*	Jones et al., 2008
Prostate and Testicle	PH	nc	*10, 17*	Li et al., 2011; Lin et al., 2014b
Stomach	PH	nc	*8, 10*	Yu et al., 2009; Zhang et al., 2012
	both	nc	*17*	Hu et al., 2013; Kang et al., 2013
	SA	c	*B1, GA9*	Kang et al., 2013; Weng et al., 2018
		nc	*7*	Chen H. F. et al., 2017

*The clustered (c) and non-clustered (nc) PCDHs are grouped.*

In contrast, some studies have correlated hypomethylation leading to ectopic expression of specific *PCDHs* with cancer progression, suggesting context-specific roles for these molecules. Examples include *PCDHB9* in gastric cancer, *PCDHGC5* in astrocytomas, and *PCDH11Y* in prostate cancer (Yang et al., 2005; Mukai et al., 2017; Vega-Benedetti et al., 2019). The mechanisms via which ectopic *PCDH* expression results in cancer progression are insufficiently investigated, however, pathological outcomes (e.g., metastasis, increased chance of relapse, drug resistance) are comparable to those observed with loss of *PCDH* expression as described above (Yang et al., 2005; Mukai et al., 2017; Vega-Benedetti et al., 2019). Intriguingly, expression of *PCDH7* and *PCDH10*, which are generally considered to be tumor suppressor genes, has been proven necessary for the tumorigenicity of non-small cell lung cancer and glioblastoma, respectively (Echizen et al., 2014; Zhou X. et al., 2017). PCDH7 induces MAPK signaling in non-small cell lung cancer, and its expression is associated with poor prognosis (Zhou X. et al., 2017). Moreover, it has been hypothesized that *PCDH11Y* expression might occur downstream of Relaxin, and its increase could drive neuroendocrine transdifferentiation by activating the Wnt signaling pathway (Yang et al., 2005; Thompson et al., 2010).

Taken together, it is clear that dysregulation of Pcdh expression is extensively linked to tumor progression. However, while several roles of Pcdhs in neuronal development have been mechanistically dissected, most oncological studies have focused on the characterization of PCDHs as potential biomarkers. The molecular processes they mediate in cancer therefore remain mostly unknown. In general, PCDHs seem to regulate cancer cell proliferation, migration and/or apoptosis. Interestingly, individual PCDH functions seem to be context-dependent, as some PCDHs can be considered both tumor suppressors or proto-oncogenes according to the type of cancer examined.

## Structural Aspects of Pcdh Interactions

To fulfill different roles in neural circuit development and cancer, Pcdhs engage in unique interactions with other cadherin motif-containing molecules. According to the classical view, Pcdhs interact homophilically in *trans* (apposing cell membranes) via their extracellular cadherin motifs. Binding in *trans* has been studied using cell aggregation assays in K562 cells. These cells are non-adherent, lack endogenous expression of Pcdhs, and provide an ideal system to study the effect of Pcdh expression on adhesion behavior in cell aggregation assays. These assays have been used to obtain qualitative readouts of *trans* interactions and to quantitatively measure binding affinity, and have allowed the discrimination between different adhesion levels and co-aggregation patterns (Schreiner and Weiner, 2010; Thu et al., 2014; Rubinstein et al., 2017; Bisogni et al., 2018). Combined results from X-ray crystallography, bioinformatic modeling, cell aggregation assays, and evolutionary correlations have further revealed that *trans* homophilic interfaces are formed between specific cadherin repeats.

Besides homophilic *trans* binding, cell aggregation assays, solution biophysical measurements and X-ray crystallography have shown that cPcdhs can engage in homotypic and heterotypic dimers on the same cell membrane via *cis* interactions, expanding the array of unique cell surface protein identities (Schreiner and Weiner, 2010; Thu et al., 2014; Rubinstein et al., 2015; Goodman et al., 2016a). Similar *cis* interactions through the extracellular domains have not been found for ncPcdhs (Harrison et al., 2020), but it cannot be excluded that other domains (transmembrane or intracellular) mediate *cis* interactions between these Pcdh members.

Both *trans* and *cis* interactions thus confer unique features (adhesion and cell surface “barcoding”, respectively), to Pcdh-expressing cells, which might explain how less than 100 isoforms create the necessary variety for self-recognition and neuronal wiring.

### *Trans* Interactions

#### Clustered Pcdhs

Pcdhs interactions differ significantly from those occurring between classical cadherins. Pcdhs are only partially dependent on calcium for *trans* binding (Schreiner and Weiner, 2010); moreover, cPcdhs lack a hydrophobic pocket and have fewer glycosylation sites (Morishita et al., 2006; Schreiner and Weiner, 2010; Thu et al., 2014; Nicoludis et al., 2015; Rubinstein et al., 2015). Analyses on γ-Pcdhs first identified the EC2 and EC3 domains (with EC1 being the most N-terminal cadherin domain) as those mediating specificity in homophilic *trans* interactions between Pcdhs (Schreiner and Weiner, 2010; Thu et al., 2014). Among cPcdhs, only PcdhαC1 was found to not conform to the homophilic adhesion rule, likely due to the absence of a calcium binding motif in EC3 which might affect its structure (Thu et al., 2014).

*Trans* homophilic interfaces are formed via EC1–EC4 antiparallel domain interactions in a head-to-tail orientation, allowing binding of EC1 with EC4, and EC2 with EC3 (Nicoludis et al., 2015). Single-domain mismatches between either EC4 and EC1 or EC2 and EC3 were shown to be sufficient in, respectively, blocking *trans* dimerization or preventing co-aggregation (Rubinstein et al., 2015).

**FIGURE 3:**
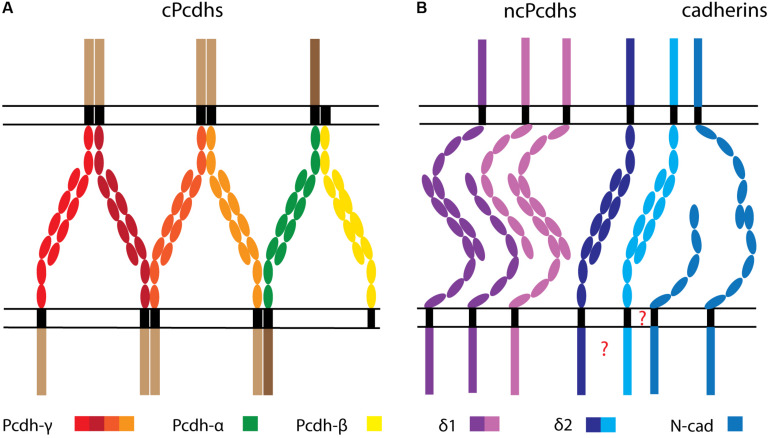
Depiction of hypothetical cPcdh-, ncPcdh-, and classical cadherin-mediated interactions between cell membranes. **(A)** cPcdhs interact to form a zipper-like array consisting of matching *cis* homo- or heteromultimers on opposed surfaces, associating in *trans* via homophilic monomer binding. **(B)** δ1-Pcdhs form *trans* homodimers and weaker *trans* heterodimers. δ2-Pcdhs also form *trans* homodimers, and might further associate with classical cadherins (e.g., N-cad), potentially through the transmembrane domain. Whether ncPcdh interact in *cis* through the transmembrane or intracellular domain is unknown.

Several studies examining the Pcdh adhesive interface furthermore revealed that both its conformation and the interaction preferences of interface-localized residues are necessary for Pcdh binding specificity (Nicoludis et al., 2015, 2016; Cooper et al., 2016; Goodman et al., 2016a, b; Brasch et al., 2019). *Trans* interaction specificity is mediated by the EC1–EC4 interface, whereas the EC2–EC3 interface contributes to a greater extent to *trans* interaction affinity (Goodman et al., 2016b; Nicoludis et al., 2019) (Figure 3).

### Non-clustered Pcdhs

δ-PCDH are also characterized by homophilic interactions in *trans* (Hoshina et al., 2013; Bisogni et al., 2018; Harrison et al., 2020). Structurally, the *trans-*dimers formed by cPcdhs and δ2-Pcdhs were proven to be quite similar. Indeed, X-ray crystallography analyses revealed that two zebrafish Pcdh19 molecules on adjacent cells interact via a “forearm handshake” involving EC1–EC4 domain binding (Cooper et al., 2016), and that human PCDH1 proteins also homophilically dimerize in an antiparallel manner through these domains (Modak and Sotomayor, 2019). Cell/bead aggregation studies using Pcdh7 EC1-4 or Pcdh9 single deletion mutants demonstrated that EC1–EC4 and EC2–EC3 interactions seem to confer, respectively, dimer binding affinity and binding specificity (Bisogni et al., 2018; Peng et al., 2018).

Moreover, aggregation assays performed on K562 cells showed that, like for cPcdhs, the combinatorial expression of δ-Pcdhs supports self-recognition; thus, cells presenting identical δ-Pcdhs surface combinations aggregate, while cells expressing different δ-Pcdhs segregate. These coaggregation experiments also revealed that combination mismatches lead to three aggregation types (“intermixing”, “interfacing”, and “segregating”) defined by a high, medium, or low number of shared cell boundaries, respectively. Furthermore, centrifugation-based aggregation assays indicated that different δ-Pcdhs have distinct adhesive affinities. These characteristics, as well as the relative surface expression of diverse δ-Pcdhs, determine the overall cell adhesive activity, which is ultimately reflected in a particular co-aggregation behavior (Bisogni et al., 2018). Interestingly, intermediate aggregation modes with incomplete Pcdh surface repertoire matching have been only reported so far for δ-Pcdhs. In line with this evidence, weak *trans* heterophilic interactions have been shown to occur within the subfamilies of human δ1- and δ2-Pcdhs, although these seem subtype-specific and more prevalent between different δ1-Pcdhs rather than δ2-Pcdhs (Figure 3) (Harrison et al., 2020).

### *Cis* Interactions

#### Clustered Pcdhs

*Cis* interactions between cPcdhs are mediated via the extracellular domain and, in contrast to *trans* associations, are highly promiscuous between distinct members of the α-, β-, and γ-Pcdh clusters (Han et al., 2010; Schalm et al., 2010; Schreiner and Weiner, 2010; Thu et al., 2014; Rubinstein et al., 2015). These interactions also ensure proper delivery of α-Pcdh isoforms to the cell surface, as *cis* binding with β- and/or γ-Pcdh through the EC6 domain is crucial in this context (Thu et al., 2014; Rubinstein et al., 2015).

Additionally, cPcdhs *cis* multimerization allows the establishment of new homophilic specificities in *trans* (Schreiner and Weiner, 2010; Goodman et al., 2016b). *Cis* multimers can form independently of *trans* interactions through EC6 domains with an affinity comparable to EC1–EC4-mediated *trans* binding (Rubinstein et al., 2015). One recent study described the crystal structure of a PcdhγB7 *cis* homodimer as a model for *cis* cPcdh interactions. This analysis demonstrated that interfacing occurs asymmetrically between EC5 and EC6 domains of one molecule and the EC6 domain of the other, and suggested that cPcdh surface expression requires dimerization. As α-Pcdhs cannot dimerize, they can only provide the EC5-6 side of the dimer interface. This likely explains why α-Pcdhs necessitate heterodimerization with a carrier Pcdh for cell surface delivery (Goodman et al., 2017).

#### Non-clustered Pcdhs

Evidence for inter-ncPcdhs *cis* binding came first from studies on *Xenopus* paraxial protocadherin (PAPC/Pcdh8). PAPC molecules were shown to form *cis* homodimers via ECD-located cysteine residues, and these interactions were proven to be essential for proper PAPC trafficking, maturation, and function (Chen et al., 2007). Pcdh19 interactions in homo- or heterodimers were also demonstrated via K562 cell assays, and *cis* binding disruption was suggested to underlie the developmental abnormalities characterizing epilepsy and intellectual disability linked to females (EFMR) (Pederick et al., 2018). However, recent structural evidence obtained from the investigation of δ-Pcdhs in solution was not able to substantiate any ECD-mediated *cis* interactions. Moreover, no conservation of *cis-*interface motifs characterizing cPcdh ECDs was found in δ-Pcdhs, thereby ruling out cPcdh-like *cis-*interactions through the ECD for δ-Pcdhs (Harrison et al., 2020). The observed *cis* interactions between Pcdh19 with other δ2-Pcdhs in K562 cells might therefore rely on transmembrane or ICD interactions (Figure 3).

### Clustered Pcdh Zipper Arrays Orchestrate Cell–Cell Interactions

*Cis* associations across cPcdhs can generate structural elements necessary for *trans* Pcdh binding, which contributes to neuronal self versus non-self discrimination. Depending on the context, the interplay of these interactions can lead to cell–cell adhesion or repulsion.

Several lines of evidence including computational modeling, cell aggregation, X-ray crystallography, and cryo-electron tomography data indicate that cPcdhs establish one-dimensional zipper-like structures by simultaneously engaging in *cis* and *trans* interactions. These complexes emerge when a cPcdh *cis* dimer binds two other dimers in trans, thereby forming a lattice unit; multiple units can then linearly assemble in long arrays to bring together adjacent cell surfaces (Rubinstein et al., 2015; Goodman et al., 2017; Brasch et al., 2019) (Figure 3A). According to this interaction mechanism model, known as the “isoform mismatch chain termination” model, one isoform mismatch within the zipper assembly is sufficient to stop the linear propagation of lattice units, and therefore impair contact-induced repulsion. Thus, this model explains how differential expression and surface display of 58 cPcdhs can sustain the molecular diversity required for neuronal barcoding, self/non-self discrimination, and self-avoidance (Rubinstein et al., 2015, 2017).

Recent evidence indicated that formation of adhesive zipper-like complexes by ncPcdhs is less likely to occur. In solution, δ-Pcdhs have been observed to generate dimers, but not oligomers. Moreover, conserved key residues required for *cis* interaction in cPcdhs (Goodman et al., 2016b, 2017) were found to be missing in δ-Pcdhs. In addition, when δ-Pcdhs ECDs were attached to liposomes to visualize intermembrane adhesion, high concentrations of *trans* dimers were observed between membranes. However, the typical ordered-lattice periodicity previously observed for cPcdhs (Brasch et al., 2019) was in this case absent (Harrison et al., 2020).

### An Overarching Interaction Model Involving Classical Cadherins and ncPcdhs

Aggregation experiments showed that K562 cells co-expressing *Pcdh7* and *Pcdhb11* either segregate or interface with *Pcdhb11*-positive cells according to the ratio of expression between the two Pcdhs. The fact that ncPcdhs can modulate the aggregation strength of cPcdhs suggested the formation of heteromeric Pcdh complexes that mediate cell recognition (Bisogni et al., 2018). Although recent structural studies did not observe any cross-family interactions between cPcdhs and δ-Pcdhs, the surface plasmon resonance binding studies used extracellular EC1-4 and might have missed *cis* interactions occurring through other domains (Harrison et al., 2020). Hence, whether fine tuning of adhesion versus repulsion via Pcdh dosage regulation observed in cells coexpressing Pcdh7 and Pcdhb11 involves *cis* interactions between these Pcdhs remains to be studied.

Adhesive properties of Pcdh are also affected by classical cadherins, and some ncPcdhs were demonstrated to bind in *cis* with these molecules, although the exact domains required for these interactions remain unknown. The *Xenopus* Pcdh8 paralog PAPC is able to regulate the adhesion activity of the classical cadherin C-cad; however, whether this function is mediated by direct *cis* binding has yet to be proven (Chen and Gumbiner, 2006). In cultured rat hippocampal neurons, the *cis* association between Pcdh8 and N-cadherin (N-cad, Cdh2) through the transmembrane domain regulates N-cad endocytosis. In addition, cadherin 11 (Cdh11) was also found to associate with Pcdh8 (Yasuda et al., 2007). Moreover, genetic dissection of neural progenitor patterning in zebrafish indicates the reliance of this process on an adhesive code formed by N-cad, Cdh11, and Pcdh19 (Tsai et al., 2019). In zebrafish, N-cad and Pcdh19 or Pcdh17 interact in cis, potentially also through the transmembrane domain. In this case, the mediator of homophilic interactions is likely to be Pcdh19, whereas N-cadherin might be required as a *cis-*cofactor to achieve Pcdh-mediated cell adhesion (Biswas et al., 2010; Emond et al., 2011).

Evidence supporting *cis* interactions between classical cadherins and cPcdhs is currently scarce. Overexpression of cPcdhs and N-cad in K562 cells was shown to result in strong aggregation mediated by *trans* homophilic binding involving either cPcdh or N-cad molecules, and provided no evidence of *cis* interactions between these groups (Thu et al., 2014). However, one co-immunoprecipitation study has revealed that γ-Pcdhs can associate with R-cadherin (Cdh4) and N-cad, suggesting that these particular interactions might occur in specific contexts (Han et al., 2010).

Figure 3 synthesizes our current knowledge on Pcdh-mediated *trans* and *cis* interactions. Figure 3A depicts the zipper array model of cPcdh interaction (Brasch et al., 2019) combined with promiscuous *cis* associations between clusters (Schreiner and Weiner, 2010; Thu et al., 2014; Goodman et al., 2017). The recently described *trans* homophilic and subfamily, dependent heterophilic binding dynamics between δ-Pcdhs (Harrison et al., 2020) are illustrated in Figure 3B together with the putative interaction of δ2-Pcdhs with N-cad. Considering all available evidence regarding the association between ncPcdhs and classical cadherins, it can be hypothesized that ncPcdhs might contact classical cadherins in a context-dependent manner (Figure 3B). Indeed, other cadherin members such as Cdh11 could participate in such a complex through interaction with N-cad (Tsai et al., 2019).

How these interactions translate to specific Pcdh-associated functions is still a matter of debate. The regulation of self-avoidance by cPcdhs has been attributed to extracellular contact-mediated repulsion. While the “isoform mismatch chain termination” model relates mismatches to smaller homophilic interaction interfaces (Rubinstein et al., 2015), this model might seem counterintuitive, as larger adhesive *trans* interactions are linked to repulsion instead of adhesion. What precisely triggers the repulsive signaling downstream of zipper-like cPcdh array formation is unknown. Besides, it is also still unclear how δ-Pcdh signaling might induce repulsion, as observed in abducens motor neurons. In this case, expression of Pcdh17 lacking its ICD caused axonal clumping, indicating that the ICD transduces the repellent signal (Asakawa and Kawakami, 2018).

In the traditional view, homophilic *trans* interaction involving cadherins, including Pcdhs, results in adhesion, which is supported by the observation that interaction with classical cadherins strengthens the adhesive character (Tsai et al., 2019). Intriguingly, cPcdh homophilic binding could translate to adhesion for specific cell types as well, as reducing cell-surface Pcdh repertoire diversity to one γ-Pcdh isoform in cortical neurons appears to stabilize matching and disrupt non-matching neuron–neuron and neuron–astrocyte contacts *in vitro* and *in vivo*, resulting in enhanced or reduced dendritic arborization, respectively (Molumby et al., 2016). In conclusion, the consequences of Pcdh-mediated interactions from a cell adhesion perspective seem highly dependent on the cellular (specific cell type) and molecular (Pcdh type and subdomain characteristics) context. The elucidation of the molecular mechanisms downstream of Pcdh interaction therefore represents an important and exciting research avenue for future studies.

## Signaling Downstream of Pcdhs

Pcdhs interact with a range of molecules to regulate diverse downstream signaling pathways. The following sections will discuss molecular subdomains of Pcdhs mediating these interactions, their known intracellular partners, and downstream signaling pathways, emphasizing the link between different molecular networks and specific Pcdh-associated functions.

### The Pcdh Cytoplasmic Tail

The ICD of Pcdhs is known to play a crucial role in the activation of downstream signaling cascades including Wnt, WAVE, apoptotic, and trafficking pathways. While some of these signaling events depend on the cooperative action of multiple Pcdhs, others seem to be unique to specific Pcdhs. The α-Pcdh constant exons generate three alternative splice isoforms with either a short (B-isoform), a long (A-isoform), or no (O-isoform) ICDs (Sugino et al., 2000). These diverse cytoplasmic domains seem to have distinct functions, as mice with downregulated or truncated A-isoforms show abnormalities in fear conditioning and spatial working memory, whereas no phenotype can be observed in mice lacking the B-isoform (Fukuda et al., 2008). Within the γ-Pcdh cluster, PcdhγC3, which possesses a shorter cytoplasmic tail, is the sole isoform able to interact with and inhibit Axin1, a Wnt pathway activator (Wu and Maniatis, 1999; Mah et al., 2016).

The cytoplasmic domains of the δ-Pcdhs differ from those of the cPcdhs and are subject to alternative splicing, which creates a larger molecular diversity in ncPcdh ICDs. Intra-exonic splicing was observed for several δ-Pcdh genes (Wu and Maniatis, 1999; Redies et al., 2005; Vanhalst et al., 2005). Alternative splicing of *Pcdh8* mRNA leads to the production of two isoforms differentially expressed in the nervous system (Makarenkova et al., 2005). In the embryonic mouse brain, isoforms with variable cytoplasmic tail lengths have been reported for Pcdh1, Pcdh7, and Pcdh11X (Sano et al., 1993; Yoshida and Sugano, 1999; Yoshida et al., 1999; Kim et al., 2007; Redies et al., 2008). Specific roles have been attributed to domains within the ICD; for instance, studies have shown that the CM2 domain of Pcdh7 mediates apoptosis in mouse primary cortical neurons, and that only the short isoform of rat Pcdh8 can induce N-cad endocytosis (Yasuda et al., 2007; Xiao et al., 2018).

Several ICD interacting proteins known to be associated with a range of signaling pathways have been identified for all types of Pcdhs (for an overview and references see Table 3). Elucidating the structures of all ICDs, their molecular interactors, and the associated downstream pathways is essential to understand the diverse functions of different Pcdhs.

**TABLE 3:** Known cytoplasmic domain interactors of Pcdhs.

Pcdh	Pathway/role	Interactor	References
α	WAVE	FAK	Chen et al., 2009
		Pyk2	
		WAVE	Chen et al., 2014
γ	WAVE	FAK	Chen et al., 2009
		Pyk2	
		PKC	Keeler et al., 2015b
	WNT	Axin1	Mah et al., 2016
	GABA	γ2-GABA_A_R	Li et al., 2012a
	Apoptosis	PDCD10	Lin et al., 2010
	Calcium signaling	CAMKP	Onouchi et al., 2015
δ1	Synaptic plasticity	PP1α	Yoshida et al., 1999; Vanhalst et al., 2005
	Differentiation	Taf1	Heggem and Bradley, 2003
	TGF-β	Smad3	Faura Tellez et al., 2015
δ2	WAVE	Nap1	Nakao et al., 2008; Tai et al., 2010; Biswas et al., 2014
		Cyfip2	Tai et al., 2010
		WAVE	Chen et al., 2014
	WNT	C2kβ	Kietzmann et al., 2012
		Nlk1	Kumar et al., 2017
	GABA	α1-GABA_A_R	Bassani et al., 2018
	Migration	Dab1	Homayouni et al., 2001
	Neurosteroids	NONO	Pham et al., 2017
	Synaptic endocytosis	TAO2β	Yasuda et al., 2007

### Proteolytic Processing of Pcdhs as a Potential Signaling Mechanism

**FIGURE 4:**
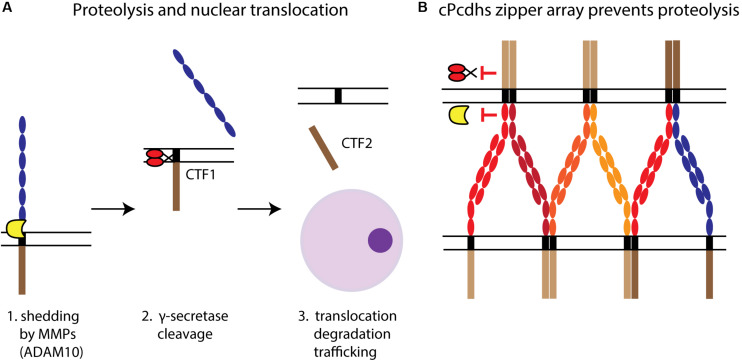
Pcdh proteolytic processing. **(A)** The proteolysis of Pcdhs and the potential translocation of their ICDs can be described as a three-step process. First, shedding by MMPs (e.g., ADAM10) releases the ECD, leaving a transmembrane C-terminal fragment (CTF1). Second, CTF1 is then cleaved by γ-secretase at the TM level to produce a soluble fragment, CTF2. Third, CTF2 (i.e., the ICD) interacts with cytoplasmic proteins and can be degraded or translocated to the nucleus, where it might participate in regulating gene expression. **(B)** cPcdhs interact at the membrane creating a zipper array characterized by alternate *cis-trans* binding. This stable complex might prevent proteolysis by making domains targeted by MMPs and γ-secretases inaccessible.

Proteolytic processing is an important transduction mechanism for type I classical cadherins, such as N-cad and epithelial cadherin (Marambaud et al., 2002; Maretzky et al., 2005; Reiss et al., 2005; Symowicz et al., 2007; Jang et al., 2011; Conant et al., 2017). ICD cleavage and subsequent nuclear translocation might also be a general characteristic of Pcdh-dependent signaling, as multiple studies have shown nuclear ICD localization for several Pcdhs (e.g., PcdhγC3, PcdhγC5, Pcdhα4, and Pcdh1α). Here, we present a brief overview of the different steps involved in the proteolytic processing and intracellular transport of the Pcdh ICD which may mediate specific Pcdh functions (Figure 4).

#### Shedding

Ectodomain shedding, whereby the ECD is cleaved by matrix metalloproteinases (MMPs) such as disintegrin and metalloproteinase domain-containing protein 10 (ADAM10) (Figure 4), was described for cPcdhs (PcdhγC3 and Pcdhα4) and ncPcdhs (Pcdh12) (Reiss et al., 2006; Buchanan et al., 2010; Bouillot et al., 2011). Additionally, shedding of PcdhγC3 and Pcdh12 was shown to be regulated by, among others, calcium-dependent and protein kinase C-mediated pathways (Reiss et al., 2006; Bouillot et al., 2011). Endocytosis prior to shedding is required for Pcdhα4, as blocking of endocytosis prevented shedding (Buchanan et al., 2010). PcdhγC3 ECD shedding by ADAM10 can be increased through the activation of glutamate receptors via α-amino-3-hydroxy-5-methyl-4-isoxazolepropionic acid (AMPA) stimulation, suggesting that neuronal activity can too regulate shedding. Furthermore, PcdhγC3 ECD shedding could be involved in the regulation of cell adhesion, as its inhibition increases cell aggregation (Reiss et al., 2006).

#### Cleavage

Pcdh ICD cleavage occurs at the TM level subsequently to shedding, and is mediated by the γ-secretase complex via its catalytic component presenilin (Reiss et al., 2006; Buchanan et al., 2010) (Figure 4A). This process might be regulated by *cis* and *trans* interactions between Pcdhs. For instance, the formation of cPcdh zipper arrays at the cell membrane could impede proteolysis by ADAM10 and γ-secretase (Figure 4B) (Hambsch et al., 2005; Brasch et al., 2019). The progressive reduction of Pcdh cleavage during neuronal differentiation, which comprises a gradual increase in Pcdh–Pcdh interactions, supports this hypothesis (Buchanan et al., 2010).

#### Nuclear Translocation

After release by cleavage, the ICD can translocate to the nucleus to regulate gene expression, be intracellularly trafficked within the endosomal system, or be degraded by the proteasome (Phillips et al., 2003; Haas et al., 2005; Fernández-Monreal et al., 2009; Hanson et al., 2010; Bouillot et al., 2011; O’Leary et al., 2011; Shonubi et al., 2015) (Figure 4A). While nuclear localization was substantiated for γ- and α-Pcdhs (Haas et al., 2005; Bonn et al., 2007; Emond and Jontes, 2008), the target genes or nuclear interactions of Pcdh ICDs are so far poorly characterized.

Regarding γ-Pcdhs, Ca^2+^/calmodulin-dependent protein kinase phosphatase (CaMPK) was shown to bind to PcdhγC5 and inhibit the nuclear translocation of the PcdhγC5 ICD. Once in the nucleus, the γ-Pcdh ICD was suggested to mediate autoregulatory γ-Pcdh expression processes by binding to the γ-Pcdh locus (Hambsch et al., 2005).

Among ncPcdhs, the ICD of human Pcdh FAT1 was found to translocate to the nucleus owing to a juxta-membrane nuclear translocation signal (NLS) (Magg et al., 2005). Moreover, the PCDH19 ICD, which contains several predicted NLSs, was shown to nuclearly localize and interact with the nuclear paraspeckle protein NONO in human cell lines (Pham et al., 2017).

#### Endosomal/Lysosomal Trafficking

Studies have shown that Pcdhs can be found in presynaptic and postsynaptic endosomal vesicles. Pcdhs can also regulate intracellular trafficking of synapse-associated proteins. For instance, Pcdh8 was demonstrated to associate with N-cad and induce its endocytosis at synapses, while Pcdh10 was shown to mediate associations between PSD-95 and the proteasome to initiate PSD-95 degradation (Yasuda et al., 2007; Tsai et al., 2012; Chal et al., 2017).

In the case of cPcdhs, evidence highlights the importance of the ICD for endosomal trafficking. For instance, the ICD of α-Pcdhs was found to interact with the endosomal sorting complex required for transport (ESCRT) in undifferentiated neuronal cells (Buchanan et al., 2010). Moreover, the ICD of γ-Pcdhs was demonstrated to be required for intracellular trafficking and cell surface delivery of these Pcdhs, and a conserved 26–residue ICD segment, known as the variable cytoplasmic domain (VCD) motif, was proven to be crucial for endolysosomal targeting (Fernández-Monreal et al., 2009, 2010; O’Leary et al., 2011; Shonubi et al., 2015). Furthermore, it was shown that colocalization of γ-PcdhA and γ-PcdhB isoforms with the endolysosomal markers autophagy protein LC3 and lysosome associated membrane protein 2 (LAMP-2) are ICD-dependent (Buchanan et al., 2010; Hanson et al., 2010). Initially, Pcdhs that do not engage in *trans* binding at the synaptic membrane were postulated to be endocytosed and stored in intracellular organelles to be eventually recycled (Phillips et al., 2003; Jontes and Phillips, 2006; Fernández-Monreal et al., 2010). Later, cPcdh trafficking was speculated to be involved in self-avoidance. According to the latter hypothesis, cPcdh-mediated matching between cell surfaces might induce endolysosomal trafficking of adhesive molecules, leading to the transition from transmembrane adhesion to detachment (Phillips et al., 2017; LaMassa et al., 2019).

### WAVE Regulatory Complex (WRC) Signaling

**FIGURE 5:**
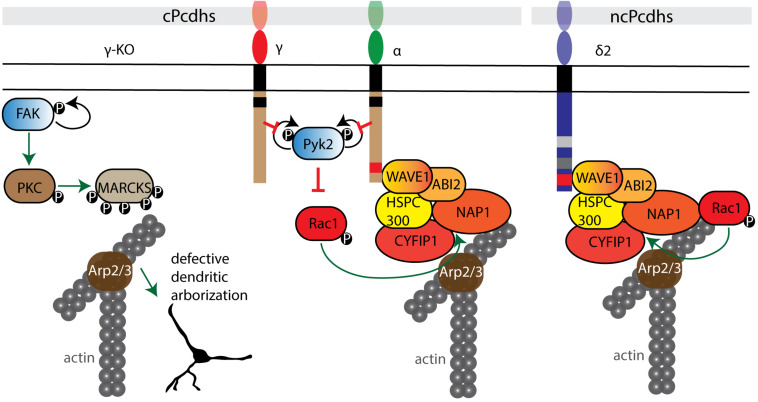
Pcdh interactions with cell adhesion kinases and the WRC. cPcdhs can regulate the WRC complex by inhibiting autophosphorylation of both tyrosine kinase Pyk2 and focal adhesion kinase FAK. In the absence of γ-Pcdhs, FAK activates by autophosphorylation and phosphorylates protein kinase C (PKC). PKC phosphorylates myristoylated alanine rich protein kinase C substrate (MARCKS), which displaces MARCKS from the cell membrane and thus impairs their actin binding activity. In the presence of cPcdhs, inactive Pyk2 cannot inhibit Rac1 phosphorylation. Subsequently, Rac1 GTPase binds the WRC complex and triggers actin polymerization via the Arp2/3 complex. cPcdhs and ncPcdhs can as well directly recruit WRC components. For instance, δ2-Pcdhs can bind to Nap1, activating GTPases that positively regulate Arp2/3-driven actin polymerization.

The actin cytoskeleton is dynamically remodeled during neurobiological processes such as neuronal migration, axon outgrowth and function, and dendritic spine formation and plasticity (Marín et al., 2010; Kevenaar and Hoogenraad, 2015; Spence and Soderling, 2015). Actin cytoskeletal dynamics are regulated by the WAVE regulatory complex (WRC), a heteropentameric complex consisting of Wiskott-Aldrich syndrome protein family verprolin-homologous protein 1 (WAVE1), cytoplasmic FMR1-interacting protein 1 (CYFIP1), Nck-associated protein (Nap1), Abelson-interacting protein 2 (Abi2) and hematopoietic stem/cell progenitor protein 300 (HSPC300); orthologs of these proteins can also function as substitute components. The WRC acts on the cytoskeleton by controlling Arp2/3 complex-mediated actin assembly (Chen et al., 2010). Several cPcdhs and ncPcdhs are involved in the activation of the WRC, which in turn stimulates the formation of F-actin (Nakao et al., 2008; Chen et al., 2009; Tai et al., 2010; Garrett et al., 2012; Suo et al., 2012; Biswas et al., 2014; Hayashi et al., 2014; Fan et al., 2018) (Figure 5).

#### cPcdhs Inhibit Cell Adhesion Kinases, and Can Recruit WRC Proteins

cPcdhs can regulate the activity of cytoskeletal regulators such as Pyk2, focal adhesion kinase (FAK), and Rho-GTPases (e.g., Rac1) in processes such as dendritic arborization (α-, γ-Pcdhs) and cortical neuron migration (α-Pcdhs) by binding through their cytoplasmic tails (Figure 5). This binding inhibits the kinase activity, thus resulting in the activation of Rho GTPases capable of modulating neuronal cytoskeletal reorganization via both WRC- and non-WRC-mediated mechanisms (Chen et al., 2009; Garrett et al., 2012; Suo et al., 2012; Fan et al., 2018).

In γ-Pcdh-deficient mice defective dendritic arborization is the result of elevated phosphorylation of myristoylated alanine rich protein kinase C substrate (MARCKS). In the absence of γ-Pcdhs, FAK is activated through autophosphorylation. FAK in turn phosphorylates and activates protein kinase C (PKC) and phospholipase C (PLC). Active PKC phosphorylates MARCKS, leading to its dissociation from the membrane and actin (Hartwig et al., 1992), and consequently to a decrease in arbor complexity (Garrett et al., 2012). Additionally, PKC can contribute to the negative regulation of dendritic arborization by phosphorylating the ICD of γ-Pcdhs and allowing FAK release (Keeler et al., 2015b).

The ICD of α-Pcdhs was found to not only regulate dendritic morphology, but also to control cortical radial migration by inhibiting the autophosphorylation of Pyk2, and by recruiting the WRC via its WAVE interacting receptor sequence (WIRS) (Chen et al., 2009; Garrett et al., 2012; Suo et al., 2012; Fan et al., 2018). This WIRS motif is present and highly conserved in several other Pcdhs, including Pcdh10, Pcdh17, Pcdh18b, and Pcdh19 (Chen et al., 2014). Aberrant dendritic development and spine morphogenesis have also been connected to loss of α-Pcdhs. When α-Pcdhs are present, Pyk2 autophosphorylation is prevented, thus Rac1 is phosphorylated and activates the WRC; ultimately, this influences the formation of lamellipodial and filopodial protrusions (Chen et al., 2009; Garrett et al., 2012; Suo et al., 2012; Fan et al., 2018).

#### ncPcdhs Recruit Nap1 and Other WRC Components

Nap1 is a core component of the WAVE complex and an important actin regulator (Chen et al., 2010). Several δ2-Pcdh ICDs (10, 17, 18b and 19) bind Nap1 through a conserved binding site, enabling δ2-Pcdh to regulate actin dynamics (Nakao et al., 2008; Tai et al., 2010; Biswas et al., 2014; Hayashi et al., 2014).

The interaction of δ2-Pcdhs with Nap1 plays a role in axon development. In deletion models, defects in axon initiation, outgrowth, pathfinding and branching have been reported in zebrafish motor neurons, mouse striatal axons, and *Xenopus* retinal ganglion cells (RGCs) (Uemura et al., 2007; Nakao et al., 2008; Piper et al., 2008; Biswas et al., 2014). The influence of these interactions on growth cone dynamics are exemplified by Pcdh17-expressing amygdala neuronal projections. Pcdh17 ICD can associate with Nap1, WAVE1 and Abi1, and recruit the WRC at inter-axonal contact sites. Additionally, Pcdh17 can recruit Lamellipodin (LPD)/MIG10 and Ena/VASP proteins via Nap1 to these sites, facilitating growth cone migration along other axons and thereby supporting collective axon extension. The GTPase Rac seems to be necessary for the recruitment of Nap1 by Pcdh17 ICD, as Rac inhibition blocks Nap1 and VASP agglomeration at contact sites (Hayashi et al., 2014). Rac is proposed to bind and regulate LPD interaction with the WRC and recruit Ena/VASP proteins to facilitate actin filament elongation (Pula and Krause, 2008; Law et al., 2013; Krause and Gautreau, 2014).

NcPcdh interactions with the WRC can also regulate cell migration processes. For instance, the Pcdh10 ICD can recruit Nap1 and WAVE1 to cell-cell contacts, and was shown to stimulate cell migration in human astrocytoma cells via F-actin and N-cad reorganization at contact sites (Nakao et al., 2008).

Thus, while cPcdhs facilitate Rho GTPases-mediated cytoskeletal reorganization principally by inhibiting cell adhesion kinases, ncPcdhs directly bind WRC components, which then promotes actin polymerization through actin regulators (Figure 5).

### Synaptic Regulatory Pathways

**FIGURE 6:**
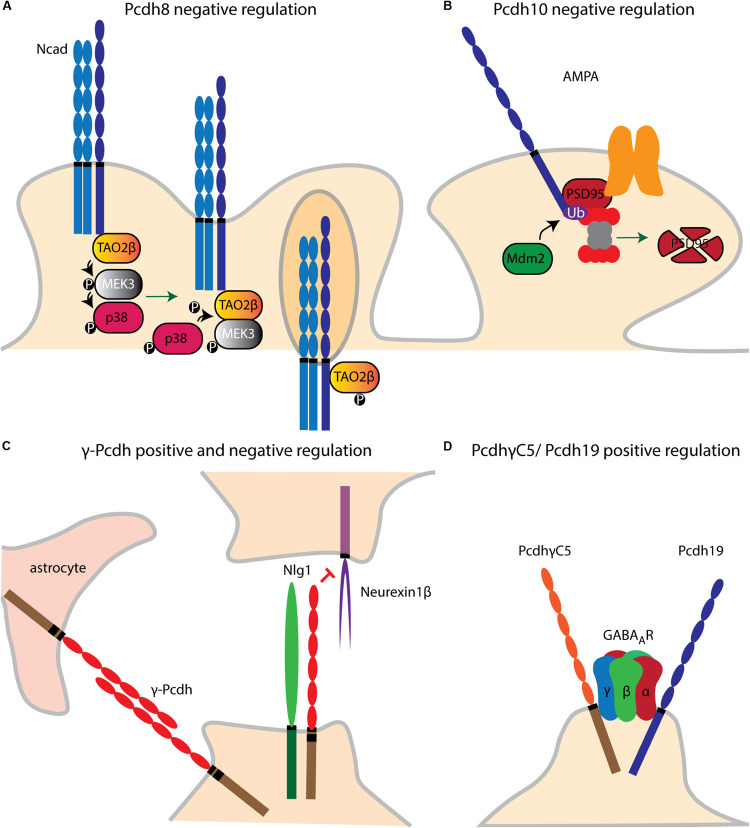
Pcdh dendritic spine and synaptic regulatory pathways. **(A)** Upon *cis* binding of Pcdh8 to Ncad, MAPK (TAO2β) is activated. A phosphorylation cascade subsequently activates MEK3 and p38. P38 phosphorylates TAO2β which results in synaptic endocytosis of Pcdh8/Ncad *cis* complex. **(B)** Pcdh10 acts downstream of MEF2 which initiates the transcription of MDM2. Once MDM2 ubiquitinates PSD-95. Pcdh10 can bind to the latter binds and associates it to the proteasome leading to synapse elimination. **(C)** γ-Pcdhs negatively regulate dendritic spine density by inhibiting the binding of Neuroligin1 (NLg1) to Neurexin1β. **(D)** The ICDs of Pcdh19 and PcdhγC5 interact, respectively, with GABA_*A*_ receptor (GABA_*A*_R) subunits α1 and γ2, respectively, and stabilize the membrane expression of the GABA_*A*_R subunits.

Pcdh8 is implicated in the control of dendritic spine density. Upon *cis* binding of Pcdh8 to N-cad, Pcdh8 ICD activates the MAP kinase (MAPK) TAO2β. This in turn activates MEK3 which then phosphorylates p38. p38 feedback signaling on TAO2β results in the synaptic endocytosis of N-cad and Pcdh8. Through this pathway, Pcdh8 was shown to downregulate the number of dendritic spines in rat hippocampal neurons (Yasuda et al., 2007) (Figure 6A).

Pcdh10 appears necessary for synapse elimination in the central nervous system. In cultured cortical and hippocampal neurons, Pcdh10 was found to act downstream of the transcription factor MEF2 to associate ubiquitinated PSD-95 with the proteasome (Tsai et al., 2012) (Figure 6B).

As mentioned above, binding of α-Pcdh ICD to Pyk2 positively regulates spine morphogenesis (Suo et al., 2012). γ-Pcdhs instead negatively regulate cortical spine morphogenesis via *cis* interaction with Neuroligin-1 (Nlg1). This binding was shown to block the interaction of Nlg1 with Neurexin1β, thus inhibiting the Nlg1-mediated presynaptic differentiation and promotion of dendritic spine density in cultured cortical neurons (Molumby et al., 2017). In contrast, another study provided evidence for positive synaptogenesis regulation by γ-Pcdhs. However, this analysis was performed in spinal cord interneurons and in combination with astrocytes, which might have contributed to synaptogenesis through Pcdh-mediated homophilic binding (Garrett and Weiner, 2009) (Figure 6C).

PcdhγC5 and Pcdh19 ICD interact in *cis* with GABA_*A*_ receptor (GABA_*A*_R) subunits γ2 and α1, respectively. Both Pcdhs regulate membrane expression of the GABA_*A*_R subunits, possibly by facilitating their trafficking to the cell surface (Li et al., 2012a; Bassani et al., 2018) (Figure 6D). Recently, a model for Alzheimer’s Disease (AD) was postulated whereby PcdhγC5 could increase inhibitory neurotransmission by enhancing synaptic GABAergic signaling and thus counterbalance the hyperexcitation caused by β-amyloid plaques (Li et al., 2017). Taken together, these findings suggest the involvement of several Pcdhs in synaptic transmission, although their specific action mechanisms in this context remain to be further examined.

### Apoptotic Pathways

#### Protocadherins as Antagonists of Oncogenic Proliferation

*PCDH10* is a tumor suppressor gene that reduces cell proliferation in hepatocellular carcinoma (HCC), and can induce cancer cell apoptosis via several routes. First, PCDH10 can negatively regulate the PI3K/Akt signaling pathway, resulting in tumor suppressor gene 53 (p53) degradation (Zhao et al., 2014; Ye et al., 2017). Second, PCDH10 can induce apoptosis by inhibiting the NF-κB pathway, thus reducing anti-apoptotic proteins such as B-cell lymphoma (Bcl)-2 and survivin (Li et al., 2014). PCDH10 expression blocks NF-κB phosphorylation and nuclear translocation via IκB kinase (IKK) inhibition, hence preventing NF-κB constitutive activation. Third, PCDH10 can directly activate caspases to trigger apoptosis (Li et al., 2014; Yang et al., 2016).

Furthermore, PCDH9 was shown to act as a tumor suppressor by eliciting apoptosis and G0/G1 cell cycle arrest in glioma cells. In these cells, PCDH9 expression, respectively, upregulated and downregulated the synthesis of BAX and BCL-2 (Wang C. et al., 2014). Similarly, in gastric cancer cells *PCDHGA9* overexpression induced apoptosis, cell cycle arrest, and autophagy. In this case, PCDHGA9 blocked TGF-β-induced epithelial-mesenchymal-transition (EMT) by inhibiting SMAD2/3 phosphorylation and nuclear translocation (Weng et al., 2018). In colorectal cancer restoring PCDH17 expression was found to enhance apoptotic pathway activation, and to induce autophagy by upregulating autophagic proteins such as Atg-5 and LC3BII (Hu et al., 2013).

In conclusion, current evidence suggests that several Pcdhs mediate pro-apoptotic functions through different signaling pathways.

#### Dosage of Pcdhs in Relation to Apoptosis in the Brain

In the developing brain both loss and overexpression of Pcdhs can elicit neuronal apoptosis. Therefore, the maintenance of proper Pcdh levels is crucial to preserve the balance between neuronal death and survival.

Excessive Pcdh7 causes primary cortical neuron apoptosis via downregulation of the apoptotic inhibitor survivin (BIRC5), and effect that is mediated by the cytoplasmic CM2 domain of Pcdh7 (Xiao et al., 2018).

Contrarily, in the absence of γ-Pcdhs Pyk2 autophosphorylates and accumulates in the cells, triggering their death (Chen et al., 2009). In addition, the γ-Pcdh ICD interacts with the intracellular adaptor protein programmed cell death 10 (PDCD10), and PDCD10 depletion attenuates chicken spinal neuron apoptosis caused by knockdown of γ-Pcdhs, implicating PDCD10 as an inducer of apoptosis downstream of these cPcdhs. In this context, γ-Pcdhs might protect neurons from apoptosis by sequestering PCDC10. Moreover, PDCD10 and Pyk2 cooperate to mediate the γ-Pcdhs-induced neuronal apoptosis (Lin et al., 2010).

Recently, γ-Pcdhs-deficient cINs were shown to have reduced phosphorylated serine-threonine kinase (AKT) levels. Numerous anti-apoptotic/pro-survival actions have been attributed to the PI3K-AKT pathway (Brunet et al., 2001), and cytoplasmic phospho-AKT is known to act as an anti-apoptotic factor. As loss of γ-Pcdhs increased apoptosis in cINs, γ-Pcdhs appear to have a role in cIN survival mediated by AKT (Carriere et al., 2020).

Overall, regulating Pcdh surface expression appears to be important for neuronal survival. Neuronal apoptosis is induced by ncPcdh overexpression or cPcdh loss. In oncogenesis, most Pcdhs seem to have a tumor suppressor function, as for several cancer types their downregulation [with the exception of PCDHB9 (Mukai et al., 2017; Sekino et al., 2019), and PCDH9 (Robbins et al., 2018)] correlates with tumor survival. Taken together, the precise dosage control of Pcdhs might therefore be crucial for cell survival in different contexts.

### Wnt Canonical and Non-canonical Signaling

Wnt signaling is a powerful regulator of cell proliferation and differentiation, and is crucially involved in cell fate determination, cellular migration, cellular polarity, organ morphogenesis, and correct tissue patterning during embryonic development (Logan and Nusse, 2004; Klaus and Birchmeier, 2008; MacDonald et al., 2009; Petersen and Reddien, 2009; Clevers et al., 2014; Sedgwick and D’Souza-Schorey, 2016; Garcin and Habib, 2017; Steinhart and Angers, 2018). Pcdhs have been mostly linked to canonical β-catenin-dependent, but also to non-canonical Wnt signaling.

#### The Relation Between Pcdh and Wnt β-catenin Signaling

**FIGURE 7:**
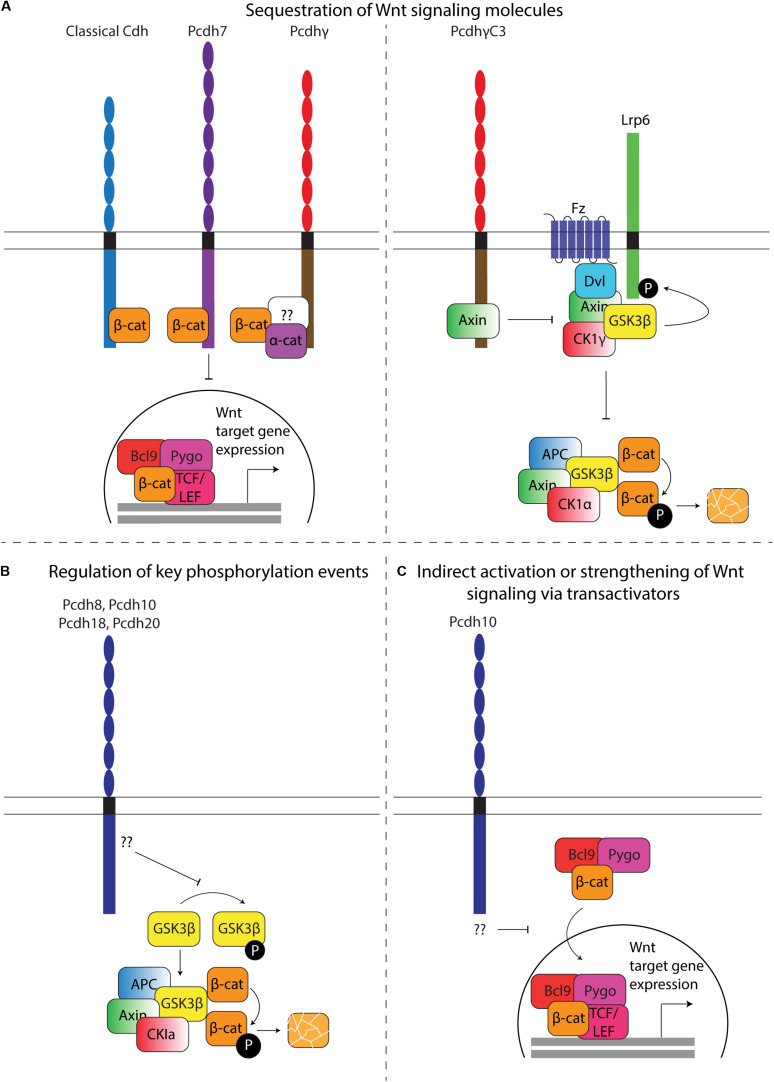
Regulation of Wnt signaling by Pcdhs. **(A)** Sequestration of Wnt signaling molecules. Left: β-catenin sequestration. Similar to classical cadherins, some Pcdhs contain a β-catenin binding site that allows retention of this molecule at the plasma membrane. A direct binding site has been identified in Pcdh7, while α- and β-catenin have been shown to co-immunoprecipitate with Pcdhγ. Whether binding occurs directly or indirectly via a common binding partner in this case is currently unknown (question mark). Sequestration results in decreased translocation of β-catenin to the nucleus, thereby reducing transcription of Wnt target molecules. Right: Axin sequestration. PcdhγC3 can directly sequester Axin, thereby competing with Disheveled (Dvl). In the absence of PcdhγC3, binding of Wnt ligands to Frizzled receptors recruits Dvl, which in turn recruits Axin. Axin can bind several kinases, including Gsk3β and Ck1γ, which activate Lrp6 by phosphorylation and inhibits β-catenin degradation. Binding of Axin to PcdhγC3 inhibits Lrp6 phosphorylation, hence indirectly stimulating cytoplasmic β-catenin degradation, and reducing transcription of Wnt target genes. **(B)** Regulation of key phosphorylation events. Overexpression of Pcdh8, Pcdh10, Pcdh18, and Pcdh20 results in an increase of active (non-phosphorylated) Gsk3β. Active Gsk3β within the destruction complex (Axin, APC, CK1a and Gsk3β) phosphorylates β-catenin, resulting in its ubiquitination and degradation by the proteasome. The mechanism through which Pcdhs regulate Gsk3β phosphorylation has yet to be characterized. **(C)** Indirect activation or strengthening of Wnt signaling via transactivators. Pcdh10 has been shown to reduce the expression of Bcl9, a β-catenin transcriptional cofactor. The Bcl9-Pygopus protein complex allows β-catenin nuclear targeting, leading to its interaction with Tcf/Lef transcription factors and ultimately the expression of Wnt target genes.

Over the last decade, evidence suggestive of a functional relationship between the Wnt signaling pathway and Pcdhs has been collected from a variety of studies. Intriguingly, the effect of Pcdh expression on Wnt signaling seems to be Pcdh- and context-dependent. The relationship between the Wnt pathway and Pcdhs has been mostly examined in cancer, where loss of Pcdhs often increases Wnt signaling, which in its turn stimulates cellular proliferation. A comprehensive overview of the different ways Pcdhs can regulate canonical Wnt signaling is shown in Figure 7.

Pcdhs appear to affect canonical Wnt signaling primarily by changing the ratio of nuclear versus cytoplasmic β-catenin. The subcellular distribution of β-catenin can be regulated at different levels. Reported mechanisms include: (1) the retention of β-catenin at the nucleus, the cytoplasm, or the plasma membrane; (2) the degradation of β-catenin in the cytoplasm by the destruction complex; (3) the independent or guided nuclear import/export of β-catenin, for example via TCF4 and BCL9 (import) or APC and Axin1 (export) (Behrens et al., 1996; Huber et al., 1996; Henderson, 2000; Neufeld et al., 2000; Rosin-Arbesfeld et al., 2000; Tolwinski and Wieschaus, 2001; Kramps et al., 2002; Cong and Varmus, 2004; Townsley et al., 2004; Krieghoff et al., 2006).

Wnt signaling regulation by β-catenin sequestration might not be limited to classical cadherins. Although it was generally accepted that all Pcdhs lack a β-catenin binding site, small serine-rich domains homologous to the β-catenin binding site of classical cadherins have been identified in the C-terminus of Pcdh7 and Pcdh11Y (Chen et al., 2002; Yang et al., 2005; Ren et al., 2018). Furthermore, mass spectrometric analysis provided direct evidence for a physical interaction between γ-Pcdhs and α- and β-catenin (Han et al., 2010).

Even without a β-catenin binding site, Pcdhs can affect the ratio of nuclear versus cytoplasmic β-catenin. *PCDH18* knockdown in a human colon mucosal epithelial cell line promotes the nuclear accumulation of β-catenin and LEF/TCF transcriptional activity, while the overexpression of PCDH10, PCDH20, or PCDHGA9 in RPMI-8226, CNE1, or SGC-7901 cells, respectively, promotes the translocation of β-catenin from the nucleus to the cytoplasm and its accumulation at the membrane, thereby decreasing LEF/TCF activity (Chen et al., 2015; Xu et al., 2015; Zhou D. et al., 2017; Weng et al., 2018). The observed negative relationship between the expression of several Pcdhs and the nuclear accumulation of β-catenin contradicts findings describing increased Wnt signaling and TCF/LEF activity due to nuclear accumulation of β-catenin with PCDH11Y overexpression in human prostate cancer cells (LNCaP) (Yang et al., 2005). This discrepancy might be related to the proto-oncogenic role of Pcdh11Y.

The mechanisms by which translocation and accumulation occurs in Pcdhs lacking a β-catenin binding site are currently unknown. Perhaps they could indirectly sequester β-catenin through a common binding partner, and regulate the release and translocation of β-catenin via phosphorylation events or ICD cleavage. One cPcdh (PCDHGC3) was found to modulate Wnt signaling by directly binding the scaffold protein Axin1 at the cell membrane (Mah et al., 2016). Thus, sequestration of Wnt signaling molecules at the plasma membrane might be common within the Pcdh family. PCDHGC3 was found to compete with Disheveled to bind the DIX domain of Axin1, resulting in its stabilization, reduced phosphorylation of LRP6 and a decrease of Wnt signaling in luciferase TOP FLASH assays (Mah et al., 2016) (Figure 7A). Intriguingly, none of the other PCDHGs can bind Axin1. In contrast, individual overexpression of other PCDHG isoforms (PCDHGA1, PCDHGA3, PCDHGA7-10, PCDHGB1-7 and PCDHGC5) was observed to significantly increase β-catenin/TCF transcriptional activity (Mah et al., 2016). Similar to *in vitro* models, *in vivo* overexpression of PCDHGA1- or PCDHGC3-mCherry in Emx1-positive cells in the murine cerebral cortex significantly increased and decreased reporter activity, respectively (Mah et al., 2016). These findings further support the hypothesized Pcdh-subtype dependent nature of the effects of Pcdh expression on Wnt signaling.

The intracellular availability of β-catenin can also be directly regulated by its degradation in the cytoplasm. Multiple studies link the overexpression or silencing of ncPcdhs (PCDH8, PCDH10, PCDH18, PCDH20) in primary tumors and tumor cell lines to reduced or increased levels of phosphorylated GSK3β, respectively (Lv et al., 2015; Xu et al., 2015; Zhou D. et al., 2017; Zong et al., 2017). These changes in phosphorylated GSK3β levels were accompanied by altered β-catenin levels and expression of Wnt target genes (Figure 7B) (Lv et al., 2015; Xu et al., 2015; Zhou D. et al., 2017; Zong et al., 2017). Pcdhs could also modulate the activity of kinases and phosphatases that play a role in Wnt signaling. For instance, signaling downstream of Fak and Pyk2 has been linked to several Wnt pathway modules (Chen et al., 2002; Gao et al., 2015, 2019; Sun et al., 2016; Zheng et al., 2019).

Finally, some Pcdhs might regulate the expression of nuclear β-catenin importers/exporters. One study described a strongly reduced expression of *BCL9* when PCDH10 was overexpressed in RPMI-8226 and KM3 cells (Xu et al., 2015). BCL9 is a necessary transcriptional co-activator of Wnt target genes, and a complex of BCL9 and Pygopus has been shown to recruit β-catenin to the nuclear compartment (Figure 7C) (Kramps et al., 2002; Townsley et al., 2004).

#### Pcdh and Non-canonical Wnt Signaling

**FIGURE 8:**
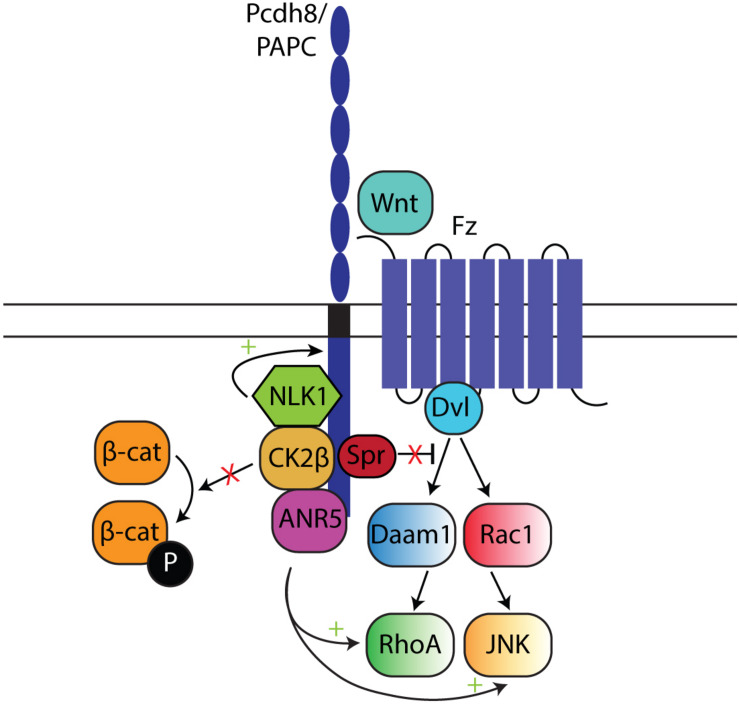
The role of Pcdh8 in Wnt/PCP signaling in Xenopus. The extracellular domain of Pcdh8 can bind Frizzled, and thus initiate the Wnt/PCP pathway. Pcdh8 sequesters Sprouty (Spr), thereby inhibiting its antagonizing effect on the Wnt/PCP pathway. In addition, Pcdh8-ANR5 interaction directly activates the effector molecules RhoA and JNK. The interaction with Nemo-like kinase 1 (NLK1) stabilizes Pcdh8, thereby ensuring continued Wnt/PCP pathway activation. Finally, the interaction with CK2β blocks a stabilizing phosphorylation of β-catenin, and ultimately results in decreased canonical Wnt signaling.

One of the best characterized Pcdh/Wnt interactions is the activation of the Wnt/Planar Cell Polarity (PCP) pathway by Pcdh8 to regulate convergent extension and tissue separation/morphogenesis during *Xenopus laevis* gastrulation (Figure 8). In the vertebrate Wnt/PCP pathway, binding of Wnt ligands to Frizzled (Fz) receptors recruits Disheveled (Dvl) to the membrane, resulting in the formation of complexes with either Disheveled-associated activator of morphogenesis 1 (Daam1) or small GTPase Rac1 that activate downstream signaling via RhoA or c-jun N-terminal kinase (JNK), respectively (Strutt et al., 1997; Axelrod et al., 1998; Boutros et al., 1998; Wallingford et al., 2000; Habas et al., 2001, 2003; Mah and Weiner, 2017). The extracellular domain of Pcdh8 is able to directly bind Frizzled7 to coordinate cellular polarity (Medina et al., 2004; Unterseher et al., 2004; Kraft et al., 2012). Both components are necessary for the initiation of Wnt/PCP signaling, as loss of Pcdh8 function was found to specifically block JNK activation via Rac1 (Unterseher et al., 2004). Furthermore, four intracellular Pcdh8 interaction partners related to the Wnt/PCP-associated molecular network have been recently discovered. The intracellular domain of Pcdh8 can sequester Sprouty to inhibit its antagonistic effect on Wnt/PCP signaling (Wang et al., 2008). Moreover, a direct physical interaction between *Xenopus* (x)ANR5 and Pcdh8 can activate the downstream effector molecules JNK and Rho, strengthening the output of the PCP pathway (Chung et al., 2007), while the interaction between Pcdh8 and Nemo-like Kinase1 (NLK1) is necessary for the stabilization of Pcdh8 (Kumar et al., 2017). Lastly, an interaction with casein kinase 2β (CK2β) blocks CK2β-mediated stabilization of β-catenin, thereby reducing canonical Wnt/β-catenin signaling (Kietzmann et al., 2012). Expression of Pcdh8 itself is regulated by Wnt/PCP signaling, providing a feedback loop into this pathway (Schambony and Wedlich, 2007).

In summary, although the majority of Pcdhs do not possess a β-catenin binding site, the interplay between cadherin-mediated adhesion and Wnt signaling seems to be conserved across the cadherin superfamily. Identifying a general interaction modality between Pcdh- and Wnt-mediated signaling pathways might prove difficult, as available evidence highlights Pcdh- and context-dependent effects on Wnt signaling. Clearly, additional research is required to better understand the complex relationship between individual Pcdhs and Wnt-related pathways. Moreover, since most studies so far have been performed in cancer cells, whether and how their findings might translate contextually to neural development is currently poorly understood. The comprehensive investigation of binding partners and molecular action mechanisms of individual Pcdhs will therefore be a crucial step in the complete elucidation of Pcdh functions across multiple contexts.

### Pyk2 and FAK Link Three Distinct Pcdh-Elicited Signaling Pathways

**FIGURE 9:**
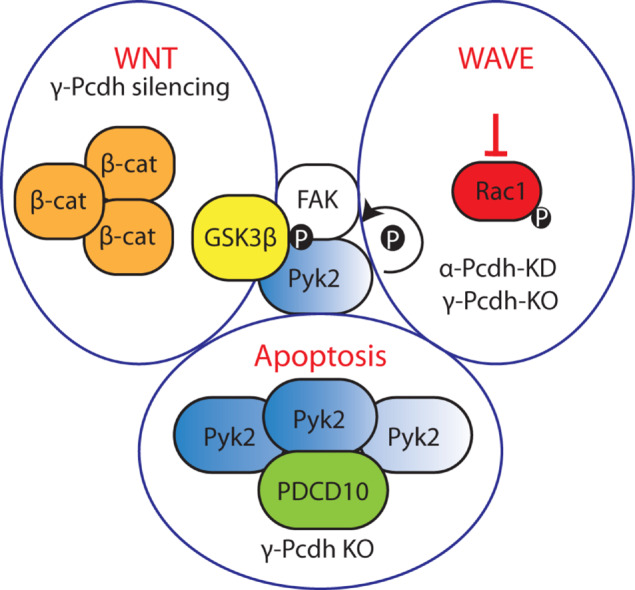
Pyk2 is a hub molecule connecting three signaling pathways modulated by Pcdhs. In cancer, silencing of Pcdhs could promote FAK/Pyk2 activation, thereby inhibiting GSK3β and activating Wnt signaling. Upon γ-Pcdh loss, Pyk2 accumulation and binding to PDCD10 elicits neuronal apoptosis. Rac1 activation and subsequent WRC complex activation is inhibited upon α- and γ-Pcdh loss due to autophosphorylation of Pyk2.

Signaling downstream of Pcdhs has been shown to also involve the Pyk2/FAK/WRC pathway. However, so far no studies have directly connected Pyk2/FAK/Wnt with Pcdh-mediated signaling, although Pyk2 and FAK are known Wnt pathway inducers. VEGF-activated FAK can directly phosphorylate β-catenin to promote Wnt signaling (Chen X. L. et al., 2012). Moreover, active Pyk2 and FAK can phosphorylate GSK3β, ultimately resulting in the degradation of GSK3β and the accumulation of β-catenin, leading to increased Wnt signaling. In addition, active Pyk2 can also phosphorylate β-catenin (Gao et al., 2015, 2019). CPcdhs regulate WRC activity by binding to Pyk2 and FAK, hence preventing their autophosphorylation and subsequent activation. Therefore, Pyk2 and FAK could represent a link between the Pcdh-regulated Wnt and WRC signaling pathways (Figure 9). Pyk2 generally acts as an oncogene, although in some cases it can function as a tumor suppressor by inducing apoptosis [reviewed in Shen and Guo (2018)].

Pyk2 has also been linked to neuronal apoptosis elicited by the absence of γ-Pcdhs. γ-Pcdh ICD negatively regulates Pyk2, preventing its autophosphorylation and Pyk2 accumulation in the cells leading to their death. Moreover, Pyk2 can interact with PDCD10 to induce apoptosis. Furthermore, γ-Pcdh ICD depletion elicits apoptosis caused by PDCD10 accumulation (Chen et al., 2009; Lin et al., 2010). Therefore, Pyk2 might represent a node connecting Wnt, WRC, and apoptotic molecular networks (Figure 9).

## Conclusion

Protocadherins play multiple roles during development, in adulthood, and in pathogenesis. In different processes the same Pcdhs can mediate opposite functions, highlighting the impact of context on Pcdh action. On a cellular-molecular level, context determines whether specific Pcdhs are expressed, inserted in the membrane, proteolytically processed, or intracellularly trafficked; in addition, it influences the availability of Pcdh interaction partners that allow the initiation of diverse cellular processes. This review aimed to provide an overview of Pcdh-driven molecular interactions and downstream signaling pathways identified within different contexts in order to identify general mechanisms of Pcdh action. In this last section, we describe major conclusions that can be derived from this survey, and potential topics for future research.

Overall, regulation of Pcdh cell surface expression is of central importance in several neurobiological processes, such as neuronal recognition or neural cell survival. In this context, the identity of Pcdhs involved in cell-cell interactions seems to particularly matter, as for instance PcdhγC4 was identified as a neuronal pro-survival isoform within the γ-Pcdh cluster (Garrett et al., 2019).

It is clear that Pcdhs are subject to proteolytic cleavage, but this process has not been systematically characterized across this protein family. Moreover, more research into the nuclear binding partners of the ICD is necessary to identify the action mechanism of Pcdhs at the nucleus and potentially directly regulated target genes. Furthermore, evidence points toward a significant endosomal recycling of Pcdhs. The extent of the influence endocytosis might have on Pcdh-mediated functions, and the Pcdh signaling modules that could be active at the endosomal level, rather than the cell membrane, are virtually unknown.

Most current knowledge on the interactions between Pcdhs and other proteins, including other Pcdhs, describes molecular mechanisms and binding dynamics at the extracellular level. Thus, many mechanistic questions regarding the interaction between intracellular proteins and the Pcdh ICD remain to be addressed. As discussed above, Pyk2 and FAK could interconnect three different Pcdh-modulated signaling pathways. Recently the PI3K-AKT pathway was shown to be involved in Pcdh-mediated neuronal survival (Carriere et al., 2020). The interplay between this pathway and Pyk2 was demonstrated in several cancer types (reviewed in Shen and Guo, 2018). Thus, Pyk2 might connect an even larger number of Pcdh-induced signaling networks. Downstream signaling through the ICD has been mostly analyzed in cancer. Despite fundamental contextual differences, interesting Pcdh-related molecular network commonalities can be identified between oncogenesis and neural development/function. Therefore, by comprehensively examining knowledge from both fields, it might be possible to gather novel insights regarding Pcdh downstream signaling pathways.

Evolutionary studies have indicated the presence of conserved motifs within the Pcdh ICD; however, their contribution to Pcdh function is not yet fully understood (Hulpiau and van Roy, 2009). Interesting to remark is that recent discoveries of independent Pcdh family expansions in animal classes evolutionarily distant from mammals, such as the Cephalopods (Albertin et al., 2015; Styfhals et al., 2019), might shed a completely novel light on Pcdh-related roles and action mechanisms, including signaling through the ICD in neural development.

Wnt and WRC pathway components have been shown to be very important mediators of Pcdh-driven functions both in brain development and in cancer. Pcdh depletion has been shown to result in neurodevelopmental defects due to a dysregulation of WRC signaling. Both cPcdhs and ncPcdhs regulate the WRC positively through the recruitment of GTPases in several contexts. Interestingly, no study has linked the WRC pathway to δ1-Pcdh functions yet. However, much less is currently known about how the absence of Pcdhs in many cancers affects WRC signaling and cytoskeletal remodeling. A hypothesis that remains to be further investigated is that increased activity of the WRC might enhance cancer cell motility, and thus malignancy.

In conclusion, the elucidation of the exact molecular mechanisms underlying the translation of Pcdh-elicited signals to the cellular machinery, as well as the components of those signaling cascades, might represent interesting and important avenues for future biomedical research. Research efforts in this direction are bound to not only increase our understanding of the mechanisms such as governing brain formation and function, but also reveal the molecular etiology of cancer.

## Author Contributions

AP wrote the most sections, and created most the figures and tables. TA produced the text, tables, and figures for the “Roles of Pcdhs in cancer” and the “Wnt canonical and non-canonical signaling” sections. MM and ES participated in reviewing and editing the manuscript’s content. All authors contributed to the article and approved the submitted version.

## Conflict of Interest

The authors declare that the research was conducted in the absence of any commercial or financial relationships that could be construed as a potential conflict of interest.
